# Loss of GalNAc-T14 links *O*-glycosylation defects to alterations in B cell homing in IgA nephropathy

**DOI:** 10.1172/JCI181164

**Published:** 2025-03-18

**Authors:** Sindhuri Prakash, Nicholas J. Steers, Yifu Li, Elena Sanchez-Rodriguez, Miguel Verbitsky, Isabel Robbins, Jenna Simpson, Sharvari Pathak, Milan Raska, Colin Reily, Anna Ng, Judy Liang, Natalia DeMaria, Amanda Katiraei, Kelsey O. Stevens, Clara Fischman, Samantha Shapiro, Swetha Kodali, Jason McCutchan, Heekuk Park, Djamila Eliby, Marco Delsante, Landino Allegri, Enrico Fiaccadori, Monica Bodria, Maddalena Marasa, Elizabeth Raveche, Bruce A. Julian, Anne-Catrin Uhlemann, Krzysztof Kiryluk, Hong Zhang, Vivette D. D’Agati, Simone Sanna-Cherchi, Jan Novak, Ali G. Gharavi

**Affiliations:** 1 Department of Medicine, Division of Nephrology, Columbia University Irving Medical Center, College of Physicians and Surgeons, New York, New York, USA.; 2 Department of Microbiology, and; 3 Department of Medicine, Division of Nephrology, University of Alabama at Birmingham, Birmingham, Alabama, USA.; 4 Department of Medicine, Division of Infectious Diseases, Columbia University Irving Medical Center, College of Physicians and Surgeons, New York, New York, USA.; 5 Departments of Medicine and Surgery, University of Parma, Parma, Italy.; 6 Department of Cell Biology and Molecular Medicine, Rutgers Biomedical and Health Sciences, Newark, New Jersey, USA.; 7 The Renal Division, Peking University First Hospital, Beijing, China.; 8 Department of Pathology and Cell Biology, Division of Renal Pathology, Columbia University Irving Medical Center, New York, New York, USA.

**Keywords:** Genetics, Nephrology, Genetic diseases, Glycobiology, Immunoglobulins

## Abstract

Aberrant *O*-glycosylation of the IgA1 hinge region is a characteristic finding in patients with IgA nephropathy (IgAN) and is thought to contribute to immune-complex formation and kidney injury. Other studies have suggested that abnormalities in mucosal immunity and lymphocyte homing are major contributors to disease. We identified a family with IgAN segregating a heterozygous predicted loss-of-function (LOF) variant in *GALNT14*, the gene encoding *N*-acetylgalactosaminyltransferase 14, one of the enzymes involved in mucin-type protein *O*-glycosylation. While *GALNT14* is expressed in IgA1-producing cells, carriers of the LOF variant did not have altered levels of poorly glycosylated IgA1, suggesting other disease mechanisms. Investigation of *Galnt14*-null mice revealed elevated serum IgA levels and ex vivo IgA production by B cells. These mice developed glomerular IgA deposition with aging and after induction of sterile colitis. *Galnt14*-null mice also displayed an attenuated mucin layer in the colon and redistribution of IgA-producing cells from mucosal to systemic sites. Adoptive-transfer experiments indicated impaired homing of spleen-derived *Galnt14*-deficient B lymphocytes, resulting in increased retention in peripheral blood. These findings suggest that abnormalities in *O*-glycosylation alter mucosal immunity and B lymphocyte homing, pointing to an expanded role of aberrant *O*-glycosylation in the pathogenesis of IgAN.

## Abstract 2

Variants in *GALNT14* were found in IgAN patients. Loss of *Galnt14* in mouse models leads to alterations in mucosal immunity and B-cell homing.

## Graphical abstract

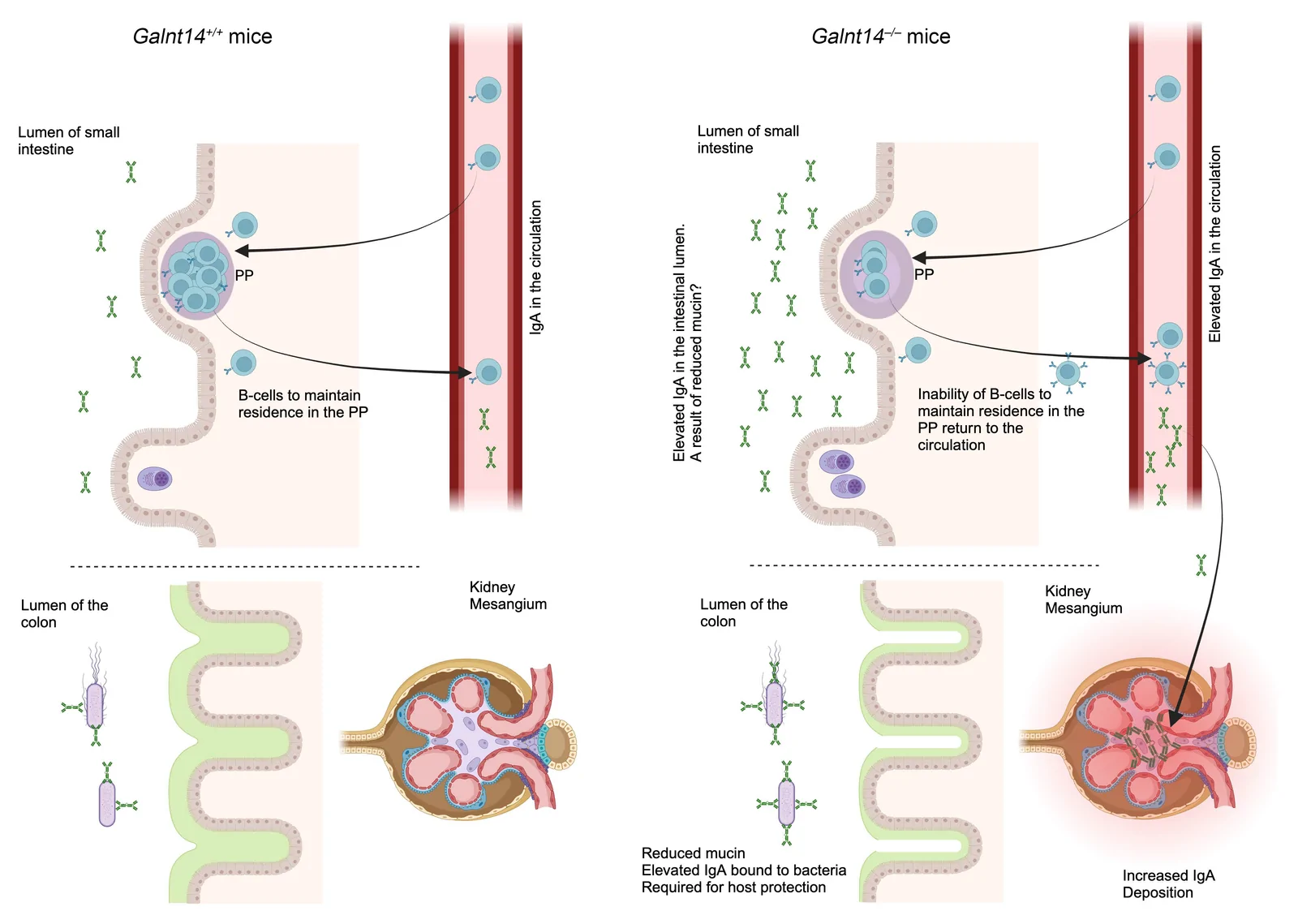

## Introduction

*O*-glycosylation is a common posttranslational modification of proteins implicated in many human disorders (1), such as cancer (2), autoimmunity (3), and the variation of triglyceride levels (4). Alterations in *O*-glycosylation of the immunoglobulin A1 (IgA1) hinge region is a salient characteristic of IgA nephropathy (IgAN), the most common primary glomerulonephritis in many countries. IgAN is a synpharyngitic nephritis; patients often present during a mucosal insult (usually an upper-respiratory–tract infection or a gastrointestinal syndrome) and develop microscopic or macroscopic hematuria and may progress to kidney failure in up to 20%–30% of cases within 10 years from presentation (5). The pathogenesis of IgAN is not fully understood. IgAN patients have characteristic IgA1-containing circulating immune complexes that deposit in the glomeruli, causing kidney injury. Multiple distinct abnormalities of the IgA system have been postulated to explain the formation of these immune complexes, including impaired *O*-glycosylation of IgA1 (6), dysregulated IgA production in response to mucosal antigens (7, 8), abnormal IgA class switching (9), and defective homing of B lymphocytes from mucosal sites to lymphoid organs (10). The temporal association of IgAN following a mucosal insult, and the finding of mucosally derived IgA1 in the serum, lymphoid organs (7, 11), and mesangial deposits (12), suggest that abnormal mucosal antigenic response and homing patterns of mucosally primed IgA1 B cells are important in the pathogenesis (13). Consistent with these findings, genome-wide association studies have uncovered many IgAN risk loci that encode genes involved in mucosal immunity, host response to pathogens, intestinal-epithelial barrier defense, and mucosal IgA production by gut-associated lymphoid tissue (14–18).

The human IgA1 has an extended hinge region with 9 serine and threonine residues (19, 20), of which usually 3–6 contain *O*-glycan modifications (21, 22). IgA1 mucin-type *O*-glycosylation occurs in a step-wise fashion, initiated by members of a family of *N*-acetylgalactosaminyltranferases (GalNAc-Ts), enzymes that add *N*-acetylgalactosamine (GalNAc) to serine or threonine residues (23). These GalNAc moieties are then galactosylated by core 1 β1,3-galactosyltransferase 1 (C1GalT1) (24), with its specific chaperone C1GalT1 specific chaperone 1 (C1GalT1C1 [also called Cosmc]) ensuring stable C1GalT1 protein expression (25). Sialic acid residues can be attached by sialyltransferases (α2,3-linked to galactose; α2,6-linked to GalNAc) (26–28). Conversely, premature addition of α2,6-linked sialic acid residues by ST6 N-Acetylgalactosaminide α-2,6-Sialyltransferase 2 (ST6GalNAc2) to GalNAc prevents galactosylation and the abbreviated glycan remains sialylated GalNAc (29–32). Patients with IgAN harbor poorly galactosylated *O*-linked glycans on the IgA1 hinge region (termed galactose-deficient IgA1; Gd-IgA1) (33). Gd-IgA1 levels are elevated in the serum of patients with IgAN and some family members with a high degree of heritability (34). Gd-IgA1 is poorly cleared by the liver, is prone to self-aggregation, and acts as an autoantigen to form immune complexes that may deposit in the glomerular mesangium leading to injury (6, 35–39). Serum levels of circulating Gd-IgA1 are associated with disease progression (40, 41). Gd-IgA1 formation is attributed to an imbalance of *O*-glycosylation enzymes in IgA1-producing cells. Patients with IgAN have decreased expression and activity of C1GalT1 (42), reduced expression of C1GalT1C1 (43), and increased expression and activity of ST6GalNAc2 (26). This process leads to either less galactosylation or premature sialylation of the GalNAc residues that blocks galactosylation (30). More recently, overexpression of one of the *N*-acetylgalactosaminyltransferases, *GALNT14*, has been shown to increase Gd-IgA1 production in the Dakiki cell line (44), a model of Gd-IgA1–producing cells that inherently overexpresses ST6GalNAc2 (26), further suggesting that dysregulation of *O*-glycosylation enzymes is an important factor in Gd-IgA1 production.

Recent studies have implicated *O*-glycosylation in many other aspects of immune regulation and IgA biology. For example, inactivation of *C1GALT1C1* in B lymphocytes results in decreased serum IgA levels, impaired homing, and reduced response to cytokines (45, 46). *O*-glycosylation is also important for maintenance of the intestinal mucus layer, as loss of *O*-glycans on intestinal mucins can increase susceptibility to spontaneous or induced intestinal inflammation (47), likely enhancing mucosal IgA production. Because *O*-glycosylation enzymes typically act on a variety of substrates across many cell types, a defect in one of them can impact multiple pathways that can coalesce and contribute to IgAN pathogenesis (48). Here, we report on the finding of rare predicted LOF variants in an *N*-acetylgalactosaminyltransferase 14-encoding gene, *GALNT14*, in a multiplex family segregating with IgAN and in a sporadic IgAN case. Detailed characterization of *Galnt14*-null mice demonstrated elevated serum IgA levels, altered IgA distribution in different tissues, and perturbations in B lymphocyte homing and glomerular IgA deposits, indicating that, beyond a role in *O*-glycosylation of IgA1 hinge region, defective *O*-glycosylation can influence multiple aspects of IgA and B cell biology relevant to IgAN pathogenesis.

## Results

### Loss of function variants in GALNT14 in familial and sporadic IgAN.

**Figure 1:**
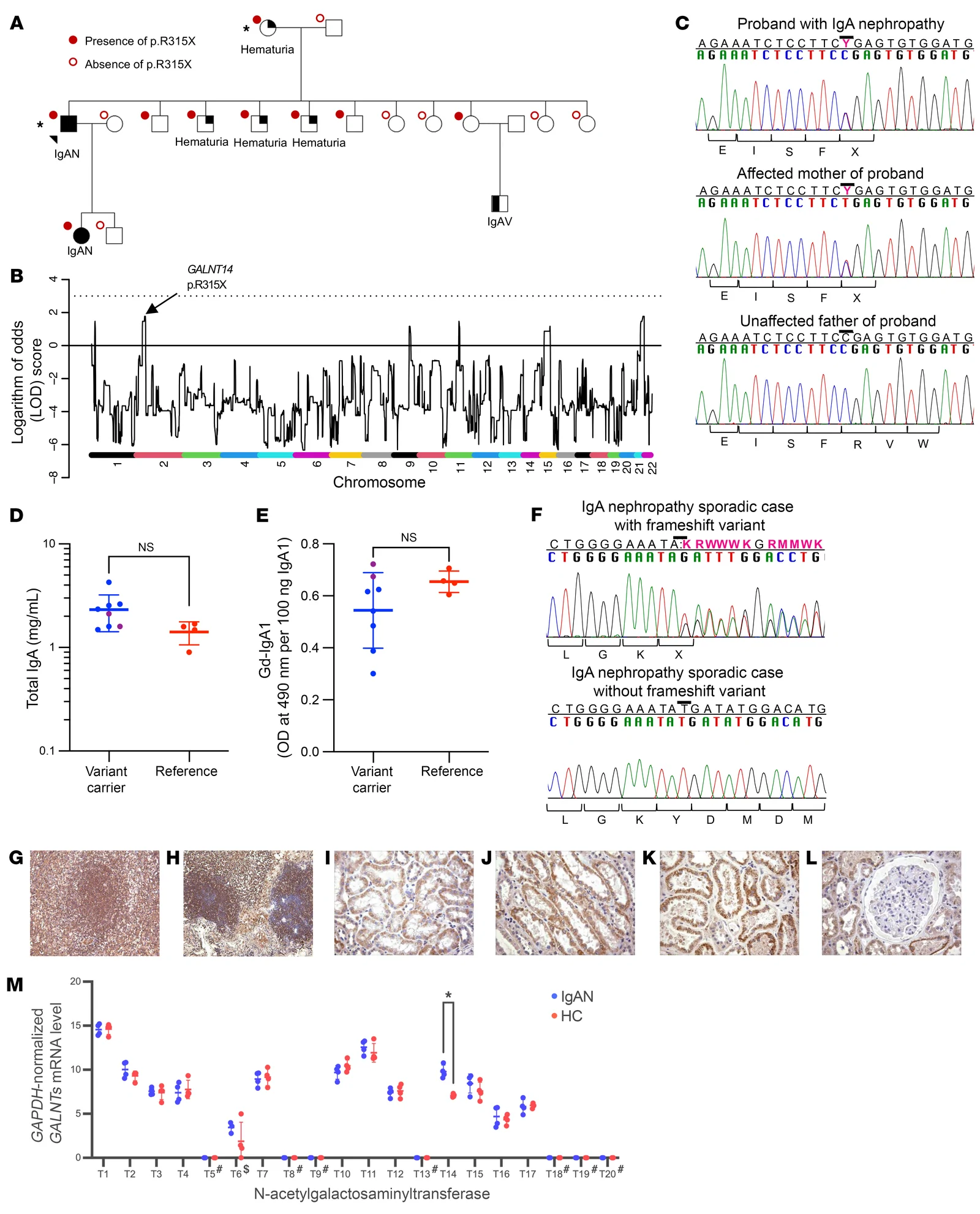
Genetic variation of *GALNT14* in familial IgAN and expression in lymphoid tissues. (**A**) A pedigree with 2 individuals with biopsy-proven IgAN (individuals with corresponding phenotype are indicated). Exome sequencing was performed in individuals with an asterisk (*) (the proband and his mother). Genome-wide genotyping was performed on all individuals with available DNA, denoted by red circles. Individuals carrying the *GALNT14* nonsense variant are denoted by a fully filled-in red dot, confirmed by Sanger sequencing. (**B**) Logarithm of odds (LOD) score plot for parametric linkage analysis under the autosomal dominant model with incomplete penetrance revealed 7 top signals (totaling about 2.4% of the genome) harboring about 1,800 genes, including the nonsense variant (p.*R315X)* found in *GALNT14*. (**C**) Confirmatory Sanger sequencing was done in all individuals with available DNA. Representative chromatograms are shown here with corresponding amino acid sequence. (**D**) No differences in the total IgA serum levels between the variant carriers and noncarriers (denoted as reference). (**E**) No differences in Gd-IgA1 levels between carriers and noncarriers of the p.*R315X* variant. (**F**) Sanger sequencing of sporadic IgAN cases of revealed an additional patient with a nonsense variant resulting in a premature termination of translation (representative chromatogram and amino acid sequence is shown). (**G**) IHC of GalNAc-T14 in the human spleen (*n* = 1), (**H**) human lymph node (*n* = 1), (**I**) human kidney cortex (*n* = 1). (**J**) human kidney medulla (*n* = 1). (**K**) human proximal and distal tubules of the kidney (*n* = 1). (**L**) Human kidney glomerulus (*n* = 1), (**M**) Comparison of the expression of different *N*-acetylgalactosaminyltransferases in immortalized IgA1-secreting cell lines demonstrates elevated expression of *GALNT14* in the cells derived from the peripheral blood of patients with IgAN (*n* = 4) compared with those from healthy individuals (*n* = 4). *P = 0.006. ^#^Expression of theses genes could not be quantitated because the crossing point (Cp) exceeded the threshold for background level (40 cycles). ^$^One Cp value exceeded the assay threshold. Original magnification, ×200 (**G** and **H**), ×400 (**I**—**L**).

We studied an Italian multiplex family segregating IgAN and persistent microhematuria in multiple generations, consistent with autosomal dominant inheritance with incomplete penetrance and variable expressivity (Figure 1A). The family had 2 biopsy-proven IgAN cases, 4 individuals with microhematuria, as well as 1 individual with IgA vasculitis (IgAV). We performed genome-wide genotyping in all available family members and exome sequencing in 2 individuals: the proband with biopsy-proven recurrent IgAN, necessitating 3 kidney transplants, and his mother, who presented with microhematuria. We conducted parametric analysis of linkage under a rare-disease autosomal-dominant model with incomplete penetrance, which identified 7 genomic regions that cosegregated with the disease status (max LOD score 1.8, Figure 1B and Supplemental Table 1; supplemental material available online with this article; https://doi.org/10.1172/JCI181164DS1). We next cross annotated these regions with the results of exome sequencing performed in the 2 individuals with IgAN in the family. We searched for rare, deleterious coding variants that mapped to these linkage intervals (defined as variants with a minor allele frequency of <0.0001, and a Polyphen score of possibly or probably damaging and Combined Annotation Dependent Depletion score greater than 20, indicating the top 1% of deleteriousness based on a large reference data set of variants; see Filtering Pipeline in Supplemental Table 2). We identified 3 variants that fulfilled these criteria: a predicted loss-of-function (LOF) variant in *GALNT14* (p.R315X) and missense variants in *AVEN* (p.N197S) and in *UNC13C* (p.S469F). No variants in collagen 4A genes were found. *GALNT14* became the prime candidate because it encodes an *N*-acetylgalactosaminyltransferase with a role in the initiation of *O*-glycosylation (Tables 1 and 2). The p.R315X in *GALNT14* was the only predicted LOF variant detected within linkage intervals and in all affected individuals (IgAN, hematuria), the obligate carrier, and 2 unaffected family members consistent with incomplete penetrance (representative sequencing chromatograms shown in Figure 1C). This variant was absent in 308 ethnically matched controls from Naples, Italy (where the family originated) and in 3,592 Italian individuals who underwent exome sequencing in a study of myocardial infarction (49). This variant was detected in only 25 individuals (and never in homozygosity) in gnomAD, a database containing sequence information from over 800,000 individuals (global MAF 1.6 × 10^–5^). We further measured the serum levels of IgA and Gd-IgA1 in the index family but did not detect differences in IgA levels or the relative degree of galactose deficiency of IgA1 between carriers of the p.R315X and noncarriers (Figure 1, D and E).

We next sequenced the coding exons of *GALNT14* in 52 probands with familial IgAN but did not identify any predicted LOF. We also searched for predicted LOF variants in *GALNT14* in 418 individuals with sporadic IgAN among 2,179 patients with chronic kidney disease (CKD) in a study evaluating the diagnostic utility of exome sequencing in CKD (50). We found a single predicted LOF variant in *GALNT14* (c.909delT, p.Y303X) in one individual with IgAN but none in the remaining 1,761 individuals in the study who did not have IgAN (Figure 1F). The patient with IgAN was diagnosed at age 26 and required kidney transplantation at age 32. There was no family history of nephropathy and parents were not available to test for variant segregation. This variant has never been reported and is absent from over 800,000 individuals from the gnomAD v4.0 database. Thus, altogether, we identified 2 *GALNT14* LOFs among 471 IgAN probands (allele frequency 0.002). We identified 7 *GALNT14* LOF variants among 9,012 well-curated exomes from self-declared healthy individuals and participants from genetic studies of neurological disorders from Columbia University (allele frequency 0.0004). This control LOF frequency is consistent with data from the gnomAD database (estimated LOF frequency 0.0008) (51, 52). We do not have access to phenotypic data in these control datasets to ascertain whether the LOF carriers have IgAN or related phenotypes, such has microhematuria and IgAV, but the approximate 5-fold–higher frequency of LOF variants among IgAN cases, together with the cosegregation with disease in the original family, indicate *GALNT14*-LOF variants may be a risk factor for disease. Assuming a *GALNT14*-LOF variant frequency of 0.0008 in controls, and an odds ratio of 5–10, we estimated that a cohort of 4,000–10,000 individuals with IgAN would be required to have 80% power to detect an association with *GALNT14* LOF variants with exome-wide significance (approximately 2.5 × 10^–6^). We therefore decided to follow up our suggestive findings by functional characterization of GalNAc-T14 and analysis of *Galnt14*-null mice.

### GALNT14 is expressed in human lymphoid tissue and immortalized IgA1-producing B cells.

In humans, there are 20 members in the *N*-acetylgalactosaminyltransferase family and they are known to have differential spatial and temporal expression patterns, capable of glycosylating both redundant and unique acceptor proteins and peptides (53). Using a human cDNA library and the GTEx database, we found that *GALNT14* is expressed in epithelia-rich tissues, such as the pituitary, esophagus, mucosal tissues, lymphoid tissues, and whole blood, with the kidney having the highest expression (Supplemental Figure 1, A and B). Interestingly, in human and murine lymphoid tissues, *GALNT14* mRNA localizes to the germinal centers (GC) of spleen and lymph nodes, which are the major sites of B cell maturation, antibody class switching, and plasma-cell proliferation (Figure 1, G and H, and Supplemental Figure 2, A and B). IHC demonstrated that the expression of GalNAc-T14 in the human kidney is limited to the proximal and distal tubules of the nephron with little or no localization in the glomerulus (Figure 1, I–L, and Supplemental Figure 2C), suggesting a role for *O*-glycosylation of mucins on epithelial surfaces of kidney tubules.

Concurrently, we characterized the expression profile of *GALNTs* in IgA1-producing cell lines using RealTime PCR. Expression analysis of the 20 well-known *GALNTs* in IgA1-producing cell lines from patients with IgAN (*n* = 4) and individuals in a healthy control group (*n* = 4), showed that *GALNT14* was the only differentially expressed *GALNT* between the 2 groups. Cells from patients with IgAN had a significantly higher expression of *GALNT14* when compared with those from healthy controls (Figure 1M, *P* = 0.006). Exome sequencing did not identify any rare variants in *GALNT14* in patients from whom the cell lines were derived.

### Galnt14^–/–^ mice have elevated IgA production.

Given that murine IgA lacks the *O*-glycosylated hinge region of human IgA1, a mouse model provides the opportunity to investigate the impact of *O*-glycosylation defects on immunomodulation, independent of the *O*-glycosylation patterns of the IgA. Toward this goal, we studied a mouse with a germline inactivation of *Galnt14* on the C57BL6/J background. Homozygous *Galnt14^–/–^* mice showed minimal to no expression of *Galnt14* levels when compared with WT mice (Supplemental Figure 3). *Galnt14*^–/–^ mice were born in expected Mendelian ratios and were healthy, fertile, and had no obvious anomalies. Gross histologic examination of the heart, lung, kidney, liver, and spleen at 3 months of age showed no overt morphological abnormalities (data not shown).

**Figure 2:**
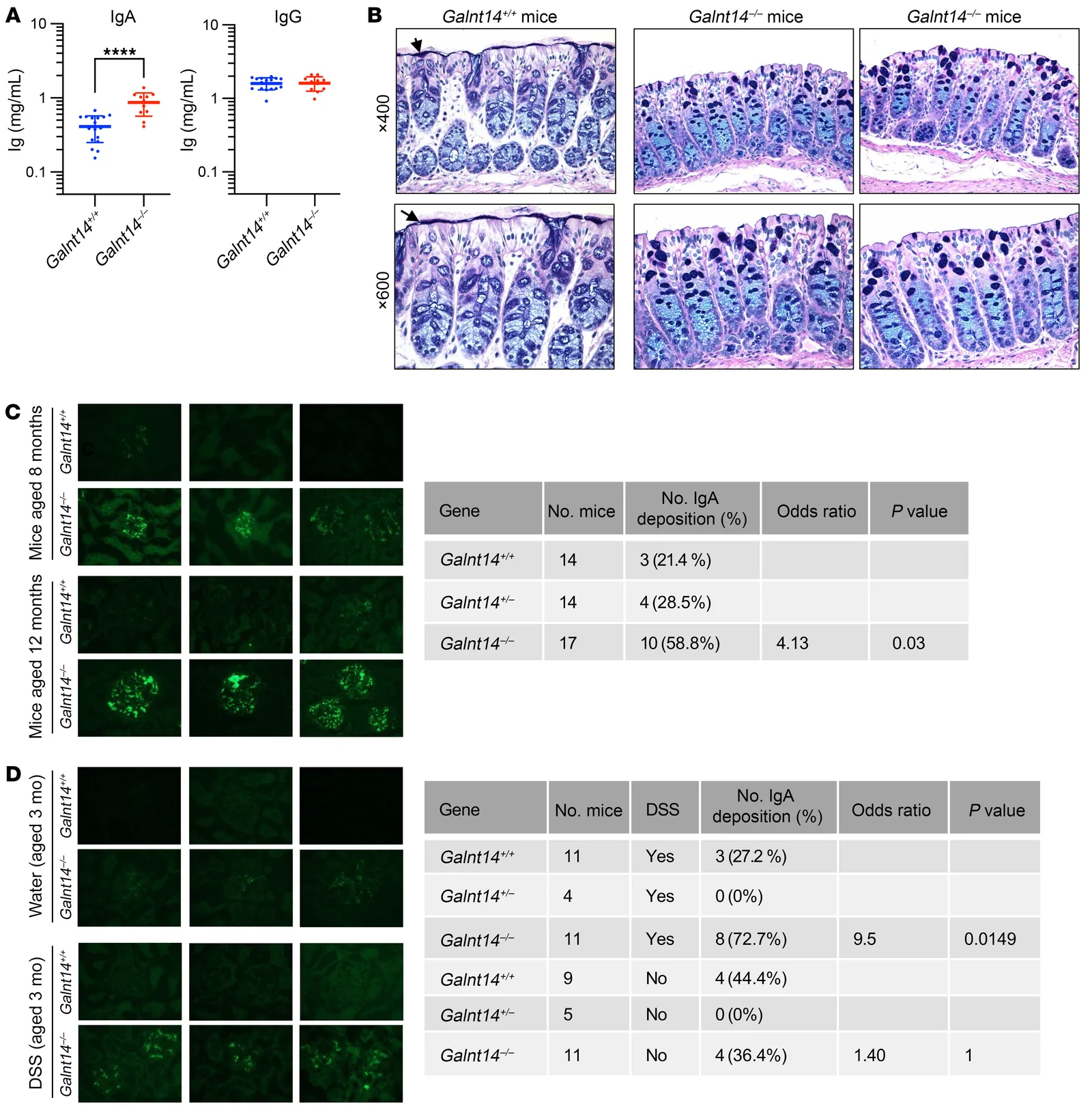
Elevated IgA in the serum and deposition of IgA in the kidneys of *Galnt14*-null mice. (**A**) Serum IgA levels were significantly elevated in *Galnt14^–/–^* mice (*n* = 11, 6 male and 5 female) compared with *Galnt14^+/+^* mice (*n* = 17, 10 male and 7 female), unpaired *t* test, ****P < 0.001. No difference was observed in the IgG serum concentrations. (**B**) Histological analysis of the colon reveals the mucin levels are reduced in the *Galnt14^–/–^* mice (*n* = 3) compared with the *Galnt14^+/+^* mice (*n* = 4). Mucin was measured at 3 points across the colon of *Galnt14^+/+^* mice (15.9 + 2.6 μm) and *Galnt14^–/–^* mice (3.2 + 0.39 μm, Supplemental Figure 5). (**C**) Mesangial IgA deposition was observed more frequently in *Galnt14*-null mice aged 8–12 months (*n* = 17, 11 male and 6 female) compared with the heterozygous and WT littermate mice (*n* = 14, 9 male and 5 female, and *n* = 14, 10 male and 4 female, respectively). (**D**) The DSS-treated *Galnt14*-null mice (aged 3 months) more frequently displayed mesangial IgA deposits (*n* = 11, 6 male and 5 female) compared with the DSS-treated heterozygous and WT littermates (*n* = 4, 2 male and 2 female, and *n* = 11, 8 male and 3 female, respectively), or water-treated mice (*Galnt14^–/–^* mice, *n* = 11, 5 male and 6 female; *Galnt14^+/–^* mice, *n* = 5, 1 male and 4 female; *Galnt14^+/+^* mice *n* = 9, 6 male and 3 female). Original magnification, ×600 (**C** and **D**).

Serum IgA levels were significantly elevated in *Galnt14*^–/–^ mice compared with their *Galnt14^+/+^* and *Galnt14^+/–^* littermates, but there was no difference in the serum IgG concentration (Figure 2A and Supplemental Figure 4). Prior studies showed that mice that lack intestinal *C1galt1^–/–^* are unable to galactosylate GalNAc or mucin *O*-glycans, leading to an attenuation of the *O*-glycan structure in the mucus layer, resulting in spontaneous colitis (54). *Galnt14* is expressed in the terminal ileum and colon in GTEx data, and IHC showed localization in the intestinal crypts, with high expression in goblet cells in the WT mouse colon (Supplemental Figure 2D). Alcian blue staining revealed that 8-week-old *Galnt14^–/–^* mice also have a reduced mucin layer in the colon, without evidence of inflammation or spontaneous colitis when compared with WT mice (Figure 2B and Supplemental Figure 5). We did not observe attenuation of the mucin layer in the kidney proximal tubule, where *Galnt14* is also highly expressed (data not shown).

### Galnt14^–/–^ mice have increased mesangial IgA deposition with aging, and with the induction of chemical colitis, in young mice.

Histopathological examination by light microscopy and IgA immunofluorescence demonstrated no evidence of glomerular injury or IgA deposition in *Galnt14*^–/–^ mice at 2–3 months of age. Given the lack of overt pathology, we tested whether stressors such as aging (55) or induction of chemical colitis (56) might alter mucosal IgA response and result in IgA mesangial deposition (57). Examining older mice, we detected increased spontaneous mesangial IgA deposition in 8- to 12-month-old *Galnt14^–/–^* mice compared with both the *Galnt14^+/–^* and *Galnt14^+/+^* mice (58.8% versus. 33.3% respectively, Fishers exact test *P* = 0.03, Figure 2C). However, no evidence of mesangial proliferation, C3 deposition, or substantial glomerular injury was appreciated. Prior studies have shown that intestinal inflammation can lead to nephritis with IgA deposition in mice (57). We therefore induced sterile intestinal inflammation with dextran sodium sulfate (DSS), resulting in rectal bleeding, diarrhea, and weight loss, as expected. Histological examination of the colon demonstrated broad sweeps of crypt erosion, diffuse lymphocyte infiltration throughout the mucosa and submucosa, epithelial cell apoptosis, necrosis, presence of crypt abscess, and evidence of epithelial cell regeneration (Supplemental Table 3). There was no genotypic difference in severity of colitis and serum IgA levels among DSS-treated animals (Supplemental Figure 6). However, 3-month-old *Galnt14^–/–^* mice treated with DSS had an increased incidence of mesangial IgA deposition when compared with both *Galnt14^+/–^* and *Galnt14^+/+^* mice (72.7% versus 20.0%, respectively, Fishers exact test, *P* = 0.0149, Figure 2D). Similar to the findings in aged *Galnt14^–/–^* mice, these kidney lesions lacked C3 codeposits. In summary, *Galnt14*^–/–^ mice demonstrated elevated serum IgA levels and mesangial IgA deposition with aging or stressors such as induction of sterile colitis.

### Elevated IgA in mucosal compartments is observed in Galnt14^–/–^ mice.

**Figure 3:**
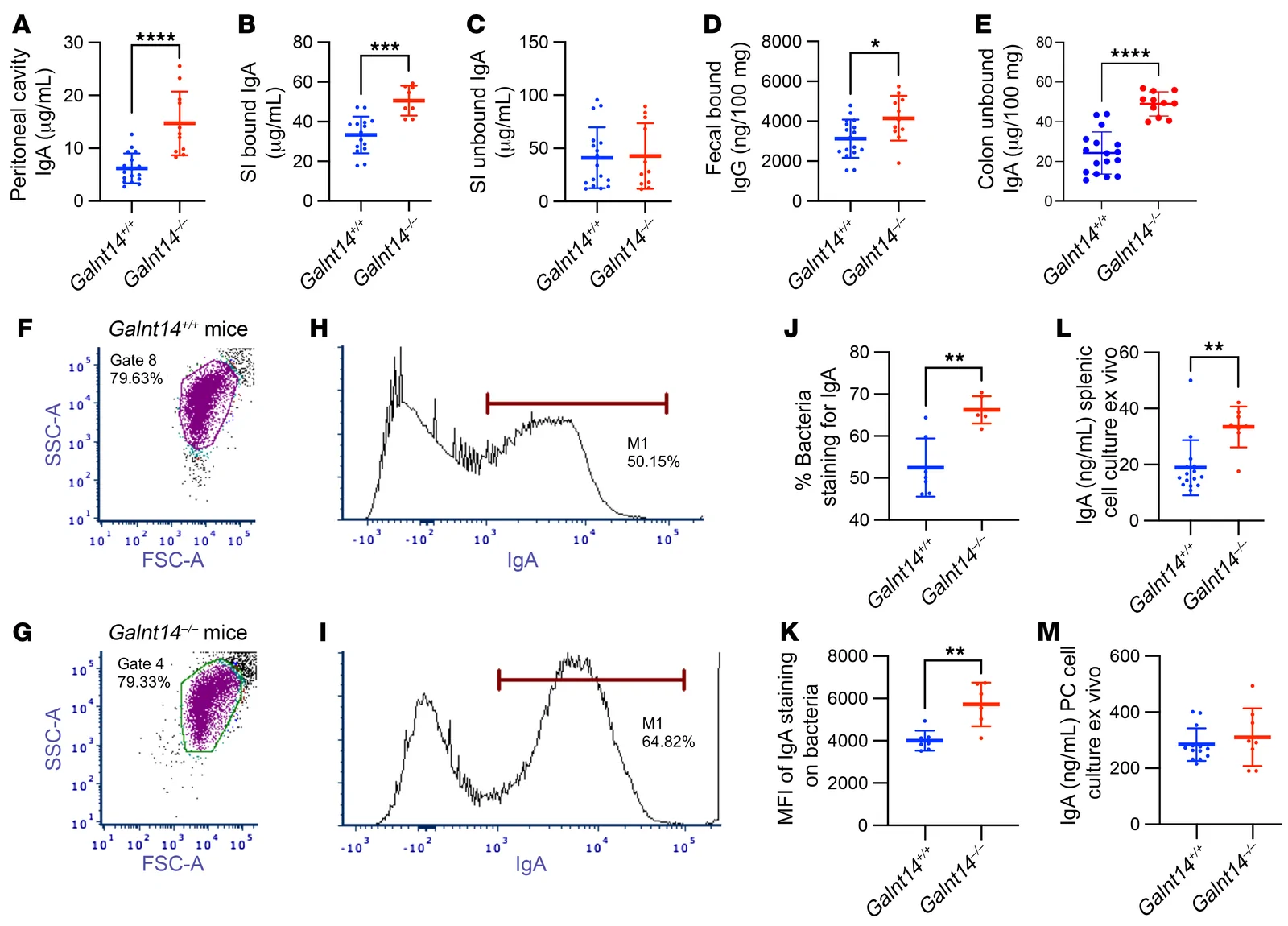
Elevated IgA concentrations in the mucosal compartments of *Galnt14^–/–^* mice. (**A**) Increased IgA in the peritoneal cavity in Galnt14^–/–^ mice (*n* = 11, 6 male and 5 female) compared to *Galnt14^+/+^* mice (*n* = 17, 10 male and 7 female) (**B**) Increased (of IgA bound to bacteria in the small intestine of the *Galnt14^–/–^* mice (*n* = 8, 4 male and 4 female) compared to the *Galnt14^+/+^* mice (*n* = 15, 9 male and 6 female). (**C**) no difference in the ‘free’ IgA in the small intestine, (**D**), increased IgA bound to fecal bacteria in the colons, (**E**) increased free IgA in the colons the *Galnt14^–/–^* mice (*n* = 11, 6 male and 5 female) compared to the *Galnt14^+/+^* mice (*n* = 17, 10 male and 7 female). (**F**) Flow cytometric analysis identifying the fecal bacteria in *Galnt14^+/+^* mice and (**G**) *Galnt14^–/–^* mice, (**H**) IgA bound to fecal bacteria in *Galnt14^+/+^* mice, and (**I**) *Galnt14^–/–^* mice. (**J**) Increased percentage of fecal bacteria identified as IgA high/positive in *Galnt14^–/–^* mice and (**K**) in *Galnt14^–/–^* mice. Mouse numbers: *Galnt14^+/+^* mice (*n* = 7, 4 male and 3 female) and *Galnt14^–/–^* mice (*n* = 6, 3 male and 3 female). (**L**) In ex vivo cultures, splenic lymphocytes from *Galnt14^–/–^* mice (*n* = 8, 4 male and 4 female) secreted significantly more IgA into the supernatant compared with splenic lymphocytes from *Galnt14^+/+^* mice (*n* = 15, 9 male and 6 female). (**M**) In ex vivo cultures of peritoneal lymphocytes, no genotype differences in the amounts of IgA secreted into the supernatant; *Galnt14^–/–^* mice (*n* = 8, 4 male and 4 female) and *Galnt14^+/+^* mice (*n* = 14, 8 male and 6 female). All comparisons are based on unpaired *t* test. *P < 0.05, **P < 0.01, ***P < 0.005, and ****P < 0.001.

We next examined the distribution of IgA levels and IgA-producing B cells in different compartments. In addition to elevated IgA levels in serum, *Galnt14^–/–^* mice had increased IgA levels, but not IgG, at multiple mucosal sites (peritoneal cavity, colon, and small intestine; Figure 3, A–E, and Supplemental Figure 7A). Bacteria-bound IgA was also increased in the small and large intestine of *Galnt14^–/–^* mice (Figure 3, B and D). Flow cytometric analysis of the fecal bacteria isolated from the colon also demonstrated enhanced IgA bound to bacteria in the *Galnt14^–/–^* mice (Figure 3, F–K, and Supplemental Table 4). In addition, ex vivo analysis of lymphocytes derived from the spleen demonstrated significantly elevated concentrations of IgA and IgG in the culture supernatants from *Galnt14^–/–^* mice compared with supernatants from *Galnt14^+/+^* mice but not in the peritoneal cavity (Figure 3, L and M, and Supplemental Figure 7, B and C).

### No differences were observed in gut microbiota between Galnt14^+/+^ and Galnt14^–/–^ mice.

**Figure 4:**
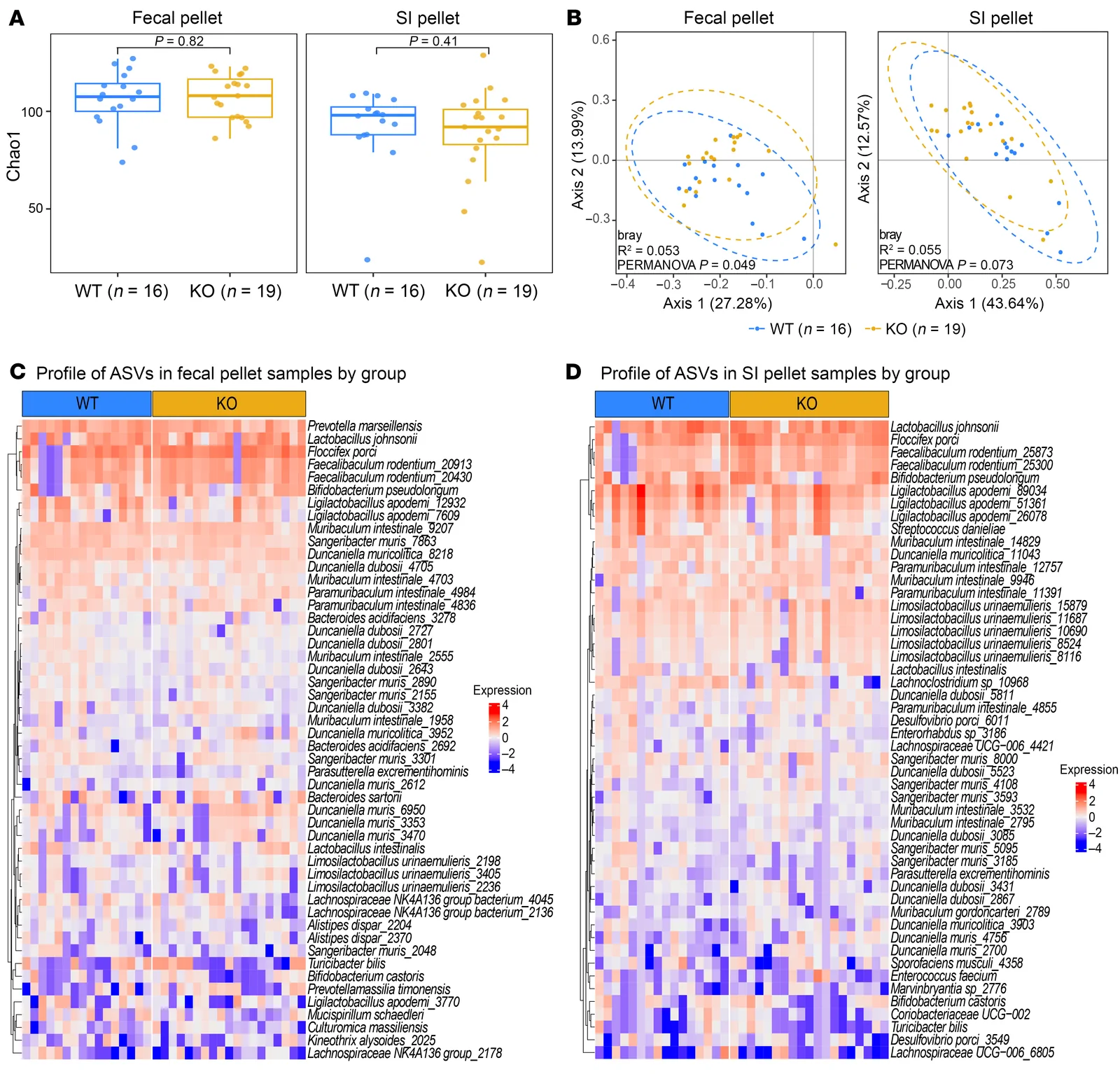
Microbiome analysis of the fecal pellets and the small intestine demonstrates no differences between the *Galnt14^–/–^* mice and *Galnt14^+/+^* mice. (**A**) α diversity comparisons (Chao1) of the fecal pellets and the small intestine revealed no differences between the *Galnt14^+/+^* mice and *Galnt14^–/–^* mice. (**B**) Principal Coordinates Analysis (PCoA) of microbial community composition across *Galnt14^+/+^* mice and *Galnt14^–/–^* mice, faceted by Fecal and Small Intestine (SI) Pellets using Bray-Curtis plots, demonstrated no differences in the β diversity. (**C**) Bacterial abundance heatmap of the fecal pellets in the *Galnt14^+/+^* mice and *Galnt14^–/–^* mice. (**D**) Bacterial abundance heatmap of the small intestine in the *Galnt14^+/+^* mice and *Galnt14^–/–^* mice. In all analyses, 16 *Galnt14^+/+^* mice (9 male and 7 female) and 19 *Galnt14^–/–^* mice (12 male and 7 female).

To determine if alteration in the microbiome accompanied attenuation of the mucin layer of the colon, we performed 16S RNA analysis of the microbiome derived from the small intestine and the fecal pellets of *Galnt14^+/+^* mice and *Galnt14^–/–^* mice. As expected, microbiome composition differed significantly between the small intestine and the fecal pellets (Supplemental Figure 9). However, the α- and β-diversity indices of the microbiome at both sites did not differ between *Galnt14^+/+^* mice and *Galnt14^–/–^* mice (Figure 4, A and B and Supplemental Figure 8). Consistent with these data, there were no differences in the relative abundance of individual microbial species between the *Galnt14^+/+^* and *Galnt14^–/–^* mice (Figure 4, C and D). These data suggest that alterations in the gut microbiome probably do not participate in the increased IgA levels and the propensity for IgA deposition in *Galnt14^–/–^* mice.

### Altered distribution of IgA^+^ B cells is observed in Galnt14^–/–^ mice.

**Figure 5:**
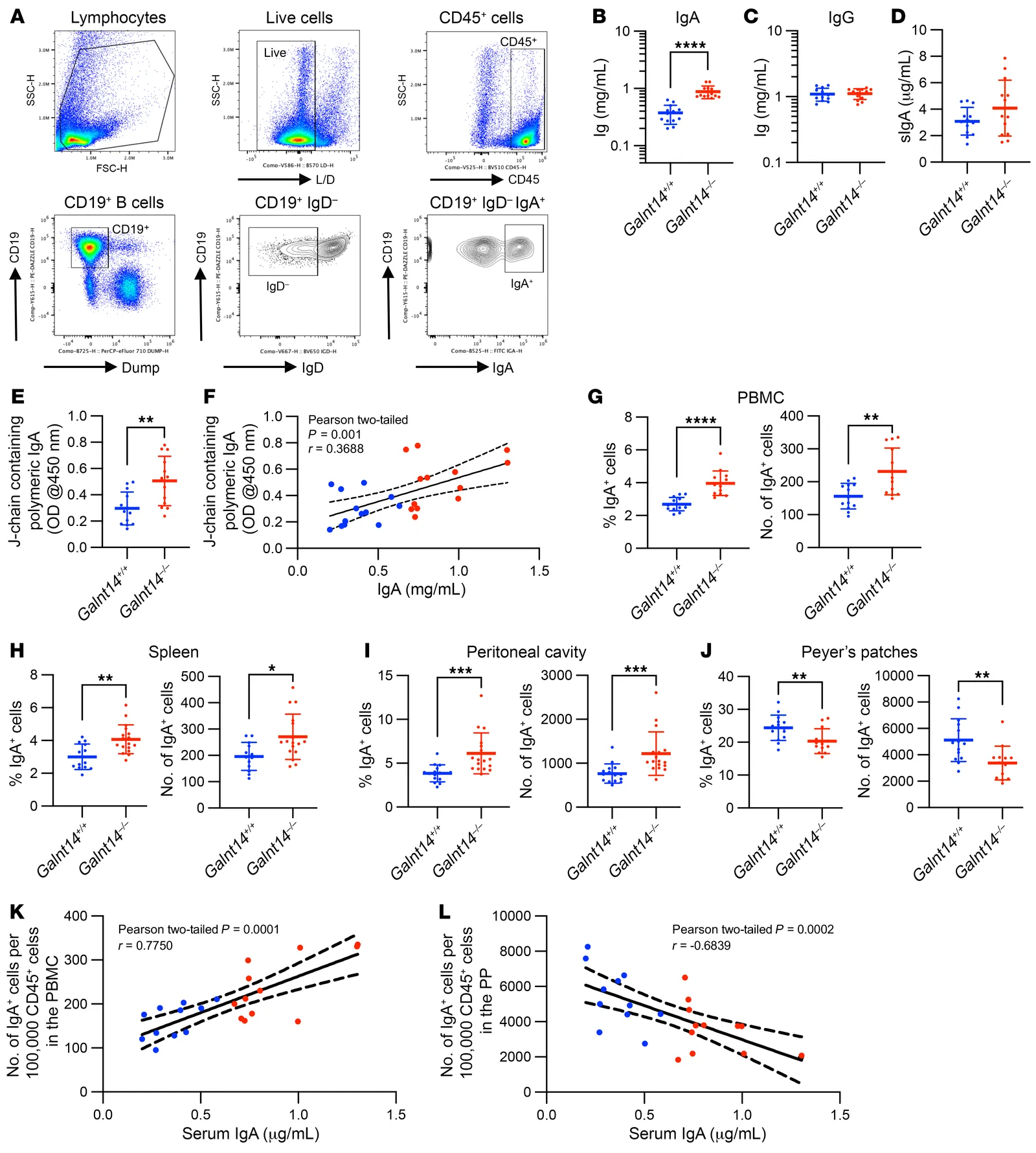
Analysis of IgA B cells in tissues of mice. (**A**) Gating strategy to identify the CD3^–^, CD14^–^, CD19^+^, IgD^–^, IgA^+^ B cells. (**B**) Increased serum levels of IgA, (**C**) no differences in serum IgG, (**D**) no differences in serum sIgA, (**E**) increased polymeric serum IgA, (**F**) A significant positive correlation was observed between serum polymeric IgA and IgA between the genotypes (*P* = 0.001, Pearson 2-tailed test, *Galnt14^–/–^* mice (*n* = 13, 6 male and 7 female) and *Galnt14^+/+^* mice (*n* = 13, 7 male and 6 female)). (**G**) A significant increase in the percentage and number of IgA^+^ B cells in the circulation of *Galnt14^–/–^* mice (*n* = 13, 6 male and 7 female) compared with *Galnt14^+/+^* mice (*n* = 13, 7 male and 6 female); (**H**) the percentage and number of IgA^+^ B cells in the spleen of *Galnt14^–/–^* mice (*n* = 17, 9 male and 8 female) compared with *Galnt14^+/+^* mice (*n* = 13, 7 male and 6 female); (**I**) the percentage and number of IgA^+^ B cells in the peritoneal cavity of *Galnt14^–/–^* mice (*n* = 17, 9 male and 8 female) compared with *Galnt14^+/+^* mice (*n* = 15, 8 male and 7 female). (**J**) A significant decrease in the percentage and number of IgA^+^ B cells in the PPs of *Galnt14^–/–^* mice (*n* = 13, 6 male and 7 female) compared with *Galnt14^+/+^* mice (*n* = 14, 7 male and 7 female). (**K**) A significant positive correlation of the number of IgA^+^ cells in the circulation with serum IgA levels (*P* = 0.0001, Pearson 2-tailed test). (**L**) A significant negative correlation of the number of IgA^+^ cells in the PP with serum IgA levels (*P* = 0.0002, Pearson 2-tailed test). *P < 0.05, **P < 0.01, ***P < 0.005, and ****P < 0.001, by an unpaired *t* test.

We further investigated the source of the elevated IgA level *in Galnt14^–/–^* mice. Using flow cytometry, we identified surface-IgA^+^ B cells in various immune tissues, including PBMCs, spleen, peritoneal cavity, Peyer’s patches (PPs), mesenteric lymph nodes (LN), mandibular LN, inguinal LN, lumbar LN, and popliteal LN in a new cohort of mice (gating strategy: Figure 5A). As before (Figure 2A), there was a significant increase in the circulating IgA level in the *Galnt14*^–/–^ mice (Figure 5B, *P* < 0.001) but no differences in the serum IgG level (5C). We also did not observe a difference in serum secretory IgA (sIgA) level between *Galnt14^–/–^* and *Galnt14^+/+^* mice (Figure 5D). Lack of elevated serum sIgA levels in *Galnt14^–/–^* mice argues against mucosal injury and a leaky gut as the source of elevated circulating IgA. On the other hand, we noted a significant difference in the J-Chain–containing IgA in *Galnt14*^–/–^ and *Galnt14^+/+^* mice (Figure 5E), indicating elevated levels of polymeric IgA in the circulation of *Galnt14*^–/–^ mice. Consistent with this finding, serum levels of J-Chain–containing IgA correlated with the levels of IgA in the circulation (Figure 5F, R^2^ = 0.3688, *P* = 0.001). There was no correlation between the serum levels of IgA and sIgA (Supplemental Figure 10A) and no correlation of serum levels of circulating IgG with the serum level of either sIgA or J-Chain–containing IgA (Supplemental Figure 10, B and C). Additionally, as expected, the greatest percentage of IgA^+^ cells were seen in PPs, consistent with the role of IgA in host defense at the largest mucosal surface in the body.

In *Galnt14^–/–^* mice, the percentage and number of IgA^+^ B cells were increased in PBMCs, spleen, and peritoneal cavity (Figure 5, G–I) and decreased in the PPs (Figure 5J). In the analysis of mucosal LNs, we observed a reduction in the percentage of IgA^+^ B cells in the mesenteric LN, and the number of IgA^+^ B cells in the mandibular LN (Supplemental Figure 10, D and E). There was no difference in the distribution of IgA^+^ B cells in the nonmucosal LNs (inguinal, lumbar, and popliteal; Supplemental Figure 10, F–H). Altogether, serum IgA levels were positively correlated with the number of IgA^+^ B cells in the PBMC (Figure 5K) and negatively correlated with the number of IgA^+^ B cells in the PPs (Figure 5L). These data suggested a redistribution of IgA-producing cells between lymph tissue and mucosal surfaces.

### Germinal center B cells in Galnt14^–/–^ mice have reduced O-glycosylation of cell surface molecules.

**Figure 6:**
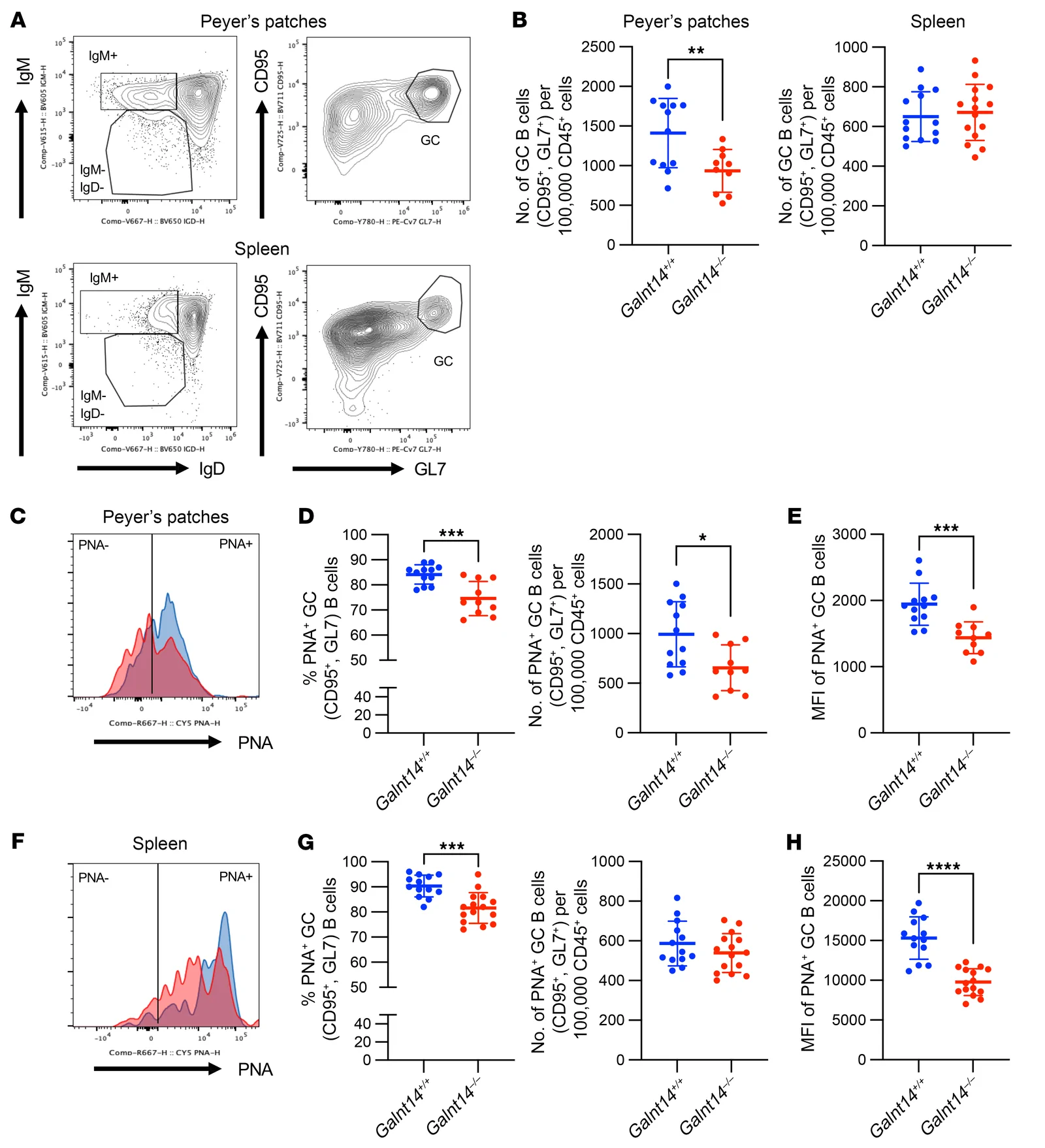
Analysis of PNA staining on the germinal center B cells in the Payer’s patches and spleens of *Galnt14^+/+^* and *Galnt14^–/–^* mice. (**A**) Identification of the GC B cells (CD19^+^, IgM^–^, IgG^–^, CD95^+^, and GL7^+^) in the PPs (top) and spleens (bottom). (**B**) A significant difference in the number of GC B cells was observed in the PPs of *Galnt14^–/–^* mice compared with *Galnt14^+/+^* mice (*P* < 0.01, unpaired *t* test). (**C**) Histogram plots of PNA staining of GC B cells in the PPs. (**D**) A significant difference in the percentage and number of PNA^+^ GC B cells was observed in the PPs of *Galnt14^–/–^* mice compared with *Galnt14^+/+^* mice (*P* < 0.01, unpaired *t* test) (**E**) A significant difference in the MFI of PNA^+^ staining on the GC B cells was observed in the PP of *Galnt14^–/–^* mice compared with *Galnt14^+/+^* mice (*P* < 0.01, unpaired *t* test (**F**) Histogram plots of PNA staining of GC B cells in the spleen of *Galnt14^–/–^* mice and *Galnt14^+/+^* mice. (**G**) No difference in the number of PNA*^+^* GC B cells was observed in the spleen; however, a significant difference in the percentage PNA*^+^* GC B cells was observed in the spleen of *Galnt14^–/–^* mice compared with *Galnt14^+/+^* mice (*P* < 0.01, unpaired *t* test) (**H**) A significant difference in the MFI of PNA+ staining on the GC B cells was observed in the spleens of *Galnt14^–/–^* mice compared with *Galnt14^+/+^* mice (*P* < 0.01, unpaired *t* test). For PP assessment, *n* = 12 (6 male and 6 female) of *Galnt14^+/+^* mice and n= 10 (5 male and 5 female) of *Galnt14^–/–^* mice. For spleen assessment, *n* = 13 (7 male and 6 female) for *Galnt14^+/+^* mice and *n* = 15 (8 male and 7 female) for *Galnt14^–/–^* mice. *P < 0.05, **P < 0.01, ***P < 0.005, and ****P < 0.001.

Prior studies have demonstrated human GC B cells express *GALNT14* and *GALNT12* and B cells from the GC express the *O*-glycosylated isoform of CD45 on the cell surface (58), which accounts for most of the staining with peanut agglutinin (PNA) lectin (Figure 6A). We therefore tested whether GC B cells in *Galnt14^–/–^* mice have a reduced *O*-glycosylation of cell surface molecules. First, we detected a global reduction of the GC B cell population in the PP and the spleen of *Galnt14^–/–^* mice (Figure 6B). Furthermore, analysis of the PNA staining of the PP showed a reduction in the PNA^+^ B cells and an overall reduction in the mean fluorescence intensity (Figure 6, C–E). In the spleen, we did not detect any changes in the total number of PNA^+^ GC B cells (Figure 6, F and G) but there was a significantly reduced percentage of PNA^+^ GC B cells and the MFI of the PNA staining (Figure 6H). It may be hypothesized that the reduction in the percentage of GC B cells in the PPs and the spleen may be due to either the inability to maintain residence in the tissue or an increased differentiation of the GC B cells into memory cells. The data suggest that the deficiency in GalNAc-T14 alters the *O*-glycosylation of cell-surface markers on B-cells in the GC of the PPs and the spleen, impacting the ability of immune cells to home and maintain residence in lymph and nonlymph tissues. PNA cell-surface staining of CD8^+^ T cells was detectable in only a small population and not affected in *Galnt14^–/–^ mice* (Supplemental Figure 11, A–C), consistent with data indicating that *Galnt14* expression is limited in T cells (44).

### Galnt14^–/–^ mice exhibit alteration in leukocyte homing.

**Figure 7:**
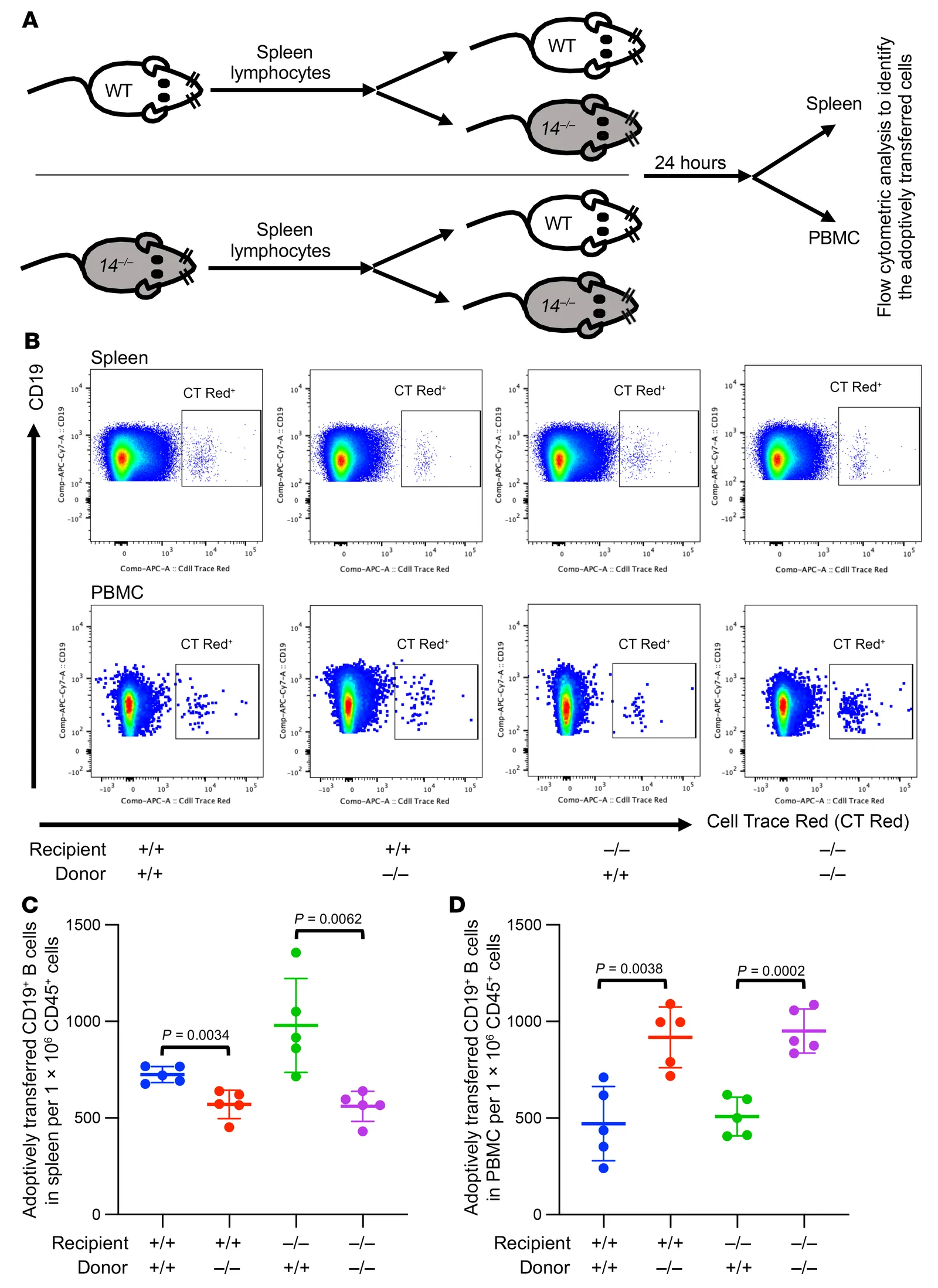
Adoptive transfer of lymphocytes from Galnt14^–/–^ mice demonstrates a deficiency in the homing ability of B cells. (**A**) Schematic of the adoptive transfer experiment. (**B**) Identification of the adoptively transferred B cells in the spleen and the peripheral blood of the recipient mice. (**C**) Adoptive transfer of 3.5 × 10^6^ lymphocytes derived from *Galnt14^–/–^* mice had significantly less (*P* < 0.01, unpaired *t* test) CD19^+^ B cells identified in the spleens of the recipient mice (*Galnt14^+/+^* or *Galnt14^–/–^*, *n* = 5 per group, 2 male and 3 female) compared with adoptively transferred lymphocytes derived from *Galnt14^+/+^* mice into recipient mice (*Galnt14^+/+^* or *Galnt14^–/–^*, *n* = 5 per group). (**D**) Adoptive transfer of 3.5 × 10^6^ lymphocytes derived from *Galnt14^–/–^* mice had increased (*P* < 0.01) CD19^+^ B cells identified in the PBMC of the recipient mice (*Galnt14^+/+^* or *Galnt14^–/–^*, *n* = 5 per group) compared with adoptively transferred lymphocytes derived from *Galnt14^+/+^* mice into the recipient mice (*Galnt14^+/+^* or *Galnt14^–/–^*, *n* = 5 per group, 2 male and 3 female).

We next tested whether the abnormal distribution of IgA-producing cells in *Galnt14^–/–^* mice may be due to altered lymphocyte trafficking. We therefore performed adoptive transfer experiments, reciprocally transferring splenic CD19^+^ B cells between *Galnt14^+/+^* and *Galnt14^–/–^* mice (Figure 7, A and B). Spleen-derived CD19^+^ B cells isolated from *Galnt14^–/–^* mice had a reduced ability to home to the spleen of recipient mice regardless of recipient genotype (either *Galnt14^+/+^* or *Galnt14^–/–^*) (Figure 7C and Supplemental Figure 12A). There was no abnormality in homing to the spleen for B cells derived from *Galnt14^+/+^* mice transferred into the *Galnt14^–/–^* recipient mice. We also did not detect differences in the homing of CD3^+^ T cells derived from either the *Galnt14^–/–^* or *Galnt14^+/+^* mice to the spleens of recipient mice (Supplemental Figure 13). Given these findings, we postulated that the number of CD19^+^ B cells derived from *Galnt14^–/–^* mice might be increased in circulation. Consistent with this hypothesis, we observed significantly more CD19^+^ B cells and CD3^+^ T cells isolated from the spleen of *Galnt14^–/–^* mice in the peripheral blood of the recipient mice compared with *Galnt14^+/+^* mice (both *Galnt14^+/+^* or *Galnt14^–/–^*, CD19^+^ cells *P* = 0.0038, *P* = 0.0002, respectively, and CD3^+^ cells *P* = 0.048, *P* = 0.002, respectively, Figure 7D and Supplemental Figure 13).

## Discussion

In this study, we implicate *GALNT14,* a gene encoding *N*-acetylgalactosaminyltransferase 14, in multiple IgAN-related phenotypes. We started by identifying independent LOF variants in *GALNT14* in individuals with familial and sporadic IgAN and detected multiple abnormalities in IgA homeostasis in *Galnt14-*null mice. GalNAc-T14 is highly expressed in human and murine lymphoid GC and in human immortalized IgA1-producing B cells. It has been previously demonstrated that GalNAc-T14 can *O*-glycosylate the IgA1 hinge region (59), and knocking down its activity might attenuate Gd-IgA1 levels from immortalized IgA1 B cells, such as the Dakiki cell line (44). However, we did not observe abnormalities in IgA1 *O*-glycosylation between variant carriers and healthy family members, leading us to explore alternative pathogenic mechanisms in a mouse model. Characterization of *Galnt14*-null mice revealed an attenuation of the intestinal mucin layer, an increase in bacteria-bound IgA in the colon, and increased susceptibility to spontaneous mesangial IgA deposition with age and following a mucosal insult. Furthermore, *Galnt14-*null mice exhibited increased levels of circulating IgA, increased ex vivo IgA production by splenic B cells, altered distribution of IgA^+^ B cells in mucosal and nonmucosal tissues, reduced *O*-glycosylation of B cell surface molecules, and impaired B cell homing to secondary lymphoid organs.

As mice only have 1 isoform of IgA that does not contain a hinge region with *O*-glycans, in vivo and ex vivo experiments in the mouse model provided the opportunity to examine alternative mechanisms through which *Galnt14* inactivation affects IgA homeostasis. *Galnt14-*null mice have reduced intestinal mucin thickness, suggesting that GalNAc-T14 participates in *O*-glycosylation of mucins in goblet cells and helps preserve the integrity of the intestinal mucosal barrier. The mucosal lymphoid tissue, such as the PPs, is a site of induction, class-switch recombination, and somatic hypermutation of B cells in the humoral immune response to produce IgA and effectively limit the penetration of mucosal pathogens into the intestinal epithelium and circulation (60–63). The elevated IgA concentration in the small intestine, colon, peritoneal cavity, and in the circulation and increased bacteria-bound fecal IgA in *Galnt14^–/–^ mice* supports the hypothesis of ongoing stimulation of the adaptive immune system and, potentially, a compensatory effort to reduce gut inflammation and bacterial translocation. The *Galnt14^–/–^* B cells from the spleen produce more IgA and IgG ex vivo, reflecting ongoing activation of B cells at mucosal surfaces or, potentially, an intrinsic defect induced by *Galnt14* inactivation. *Galnt14^–/–^* mice are also susceptible to spontaneous mesangial IgA deposition with aging, which could be due to continued priming of the adaptive immune system due to the reduction in the mucin barrier combined with the gradual age-related impairment of the mucosal barrier (55). With the induction of intestinal inflammation using DSS (a model of human inflammatory bowel disease [IBD]), mesangial deposition of IgA was observed in younger *Galnt14^–/–^* mice. These findings are consistent with the observation that patients with IBD have a high number of IgA-producing B cells (64), increased serum IgA levels, and systemic immune activation contributing to several inflammatory diseases including IgAN, which is most common glomerulonephritis in patients with IBD and is associated with progression of kidney disease (65, 66). While the human genetic data led us to investigate a LOF mechanism in the mouse model, we detected increased *GALNT14* expression in immortalized IgA-producing B cells from 4 patients with IgAN without coding variants in *GALNT14*. It is not clear whether the increased expression detected in this context is a primary defect or a reaction to other initiating events. These data motivate further investigation into the role of *GALNT14*, including gain-of-function mechanisms, in B cell homeostasis and IgA production.

We did not detect an increase of IgA-producing B cells in PPs of *Galnt14^–/–^* mice, which might be expected as a response to the attenuation of the mucin layer. In contrast, we observed reduced number of GC B cells and reduced PNA staining in PP and increased IgA B cells in the peritoneal cavity, spleen, and peripheral blood, suggesting alterations in lymphocyte homing. Consistent with this hypothesis, *Galnt14^–/–^* B cells exhibited defects in homing to lymphoid tissues. In adoptive transfer experiments, *Galnt14^–/–^* B cells derived from the spleen did not home appropriately in *Galnt14^+/+^* mice, but *Galnt14^+/+^* B cells homed correctly in recipient *Galnt14^–/–^* mice, indicating an intrinsic defect in *Galnt14^–/–^* B cells. Retention of IgA^+^ B cells in the circulation explains the positive correlation of serum IgA levels with the number of IgA^+^ B cells in the PBMC and their negative correlation with the number of IgA^+^ B cells in the PP. These data are also consistent with the reports of abnormal B cell homing after B cell–specific inactivation of *C1galt1c1* (a.k.a. *Cosmc*) (45). *Cosmc*-deficient B lymphocytes display reduced homing to lymph nodes and nonlymphoid tissues, attributable to impaired transendothelial migration and impaired response to cytokines. However, in contrast to *Galnt14*-null mice, serum levels of IgA were reduced and IgG2 levels were increased (45). Similarly, inactivation of *Galnt1* encoding another GalNAc-T expressed in lymphocytes, resulted in impaired B cell homing, with the mice exhibiting elevated IgG levels and normal IgA levels (67). The reasons for differences in immunoglobulin profiles between these mouse models may be due to the redundancy of *O*-glycosylation machinery. Our data indicate that many GalNAc-Ts have appreciable expression in IgA-producing cell lines, suggesting an extensive potential for interactions and/or compensatory changes. Alterations in the activity of GalNAc-Ts in B cells or in homing tissue can modify the GalNAc acceptor sites for C1GalT1 and ST6GalNAc2 and substantially affect protein *O*-glycosylation patterns and downstream phenotypes, as demonstrated by the reduction of PNA staining on GC B cells in PPs and spleen of *Galnt14^–/–^* mice. Genome-wide association studies have identified many cytokine pathways in susceptibility to IgAN and variation in IgA levels (68) and mice deficient in glycosylation enzymes exhibit altered responses to cytokines in vitro (69). Hence, differentially glycosylated chemokines and cytokines may also contribute to the abnormal homing patterns in these mice (45, 67, 70). At the molecular level, the mechanisms responsible for abnormal homing of *Galnt1-*, *Galnt14-*, or *Cosmc-*deficient B lymphocytes is not known. Like other investigators (45, 67), we hypothesize that defective *O*-glycosylation impacts the function of yet unknown receptor(s) or ligand(s) required for B cell homing to lymphoid tissue. The abnormal PNA staining of B cell surface in PPs and spleen supports this possibility.

Prior studies have shown 5%–10% kidneys in autopsy or donor biopsy series have IgA deposition without evidence of inflammation or histopathological evidence of IgAN. Donors with mesangial IgA deposition have higher rates of hypertension and a higher frequency of early transplant rejection. Altogether, these findings suggest that IgA deposition can become clinically important (71). Similarly, *Galnt14-*null mice exhibited mesangial IgA deposition without other clinical or histopathologic findings of IgAN but, upon further investigation, had abnormalities of IgA homeostasis. Hence, the *Galnt14-*null mice may be an appropriate model for studying the mechanisms of mesangial IgA deposition, which is considered an initiating mechanism in IgAN. Similarly, the presence of LOF variants in approximately 1:1,250 apparently healthy individuals in the control group suggests that *GALNT14* haploinsufficiency may constitute a risk factor for IgAN but may not be sufficient to cause disease. The development of IgAN and variation of IgA serum levels may depend on environmental factors such as commensal flora. We did not identify major alterations in gut microbiota in *Galnt14^–/–^* mice in a standard barrier facility but genotypic differences may be uncovered under barrier-free conditions (72). The relationship between variants in genes for the *O*-glycosylation pathways, alterations in gut microbiota, variations in serum and mucosal IgA levels, and propensity for IgA deposition will require additional investigation.

Altogether, our data indicate a role for GalNAc-T14 in IgA biology, through the unexpected defect in B lymphocyte homing. These data suggest that, beyond their impact on the IgA1 hinge region, *O*-glycosylation defects can affect additional pathways relevant to IgAN pathogenesis and potentially provide a unifying explanation for multiple abnormalities detected or posited in IgAN. Particularly, these findings encourage new areas of investigation into the role of *O*-glycosylation in the regulation of the gut mucin layer, as well as the development, localization, and homing of B cells to mucosal and nonmucosal lymphoid tissues. In addition, identification of the homing molecule(s) that are impacted by aberrant *O*-glycosylation may provide critical insight into IgAN pathogenesis.

## Methods

Full methods are available in Supplemental materials.

### Sex as a biological variable

In genetic and immunological studies, sex was considered a biological variable. We have included both males and females in the recruitment in the genetic studies in humans, and both male and female mice were included in the mouse immunology studies.

### Data availability

Values for all data points in graphs are reported in the Supporting Data Values file. Further requests for data should be directed to the corresponding authors.

### Statistics

Graphs and statistics were done in Prism (V10 for macOS). The data was initially analyzed for the distribution (normal or not normal) and statistical differences between 2 groups was identified using an unpaired 2-tailed *t* test, or a 2-tailed Mann-Whitney test. A statistically significant correlation was determined using Pearson 2-tailed test. *P* less than 0.05 was considered to be statistically significant. Data in scatter plots represents the mean ± the SD.

### Study approval

#### Human participants.

The study protocol was approved by the Institutional Review Board at Columbia University Irving Medical Center, New York. Signed written informed consent was obtained from all study participants.

#### Rodents.

All animal use was conducted in accordance with the National Institutes of Health guidelines, and the study was approved by the Institutional Animal Care and Use Committee, at Columbia University Irving Medical Center, New York, and conducted in strict accordance with recommendations in the Guide for the Care and Use of Laboratory Animals.

## Author contributions

S Prakash performed the genetic experiments and statistical genetic analysis, interpreted the genetic data, established the mouse colony, performed the aging and DSS experiments, and wrote the manuscript. NJS conceived and performed the immunological experiments, analyzed, and interpreted the data, performed statistical analysis, and wrote the manuscript. S Prakash and NJS share first authorship in the given order based on their relative contributions to the project. YL, ESR, and MV performed and analyzed the genetic experiments and interpreted the genetic data. IR, JS, S Pathak, AN, JL, ND, AK, KOS, SK, and JM performed the immunology experiments. CF performed the genetic experiments. SS performed the initial mouse experiments. MR performed the expression analysis of *GALNTs* in IgA1-secreting cell lines. CR characterized the cell lines and performed IgA and Gd-IgA1 assays. HP, DE, and ACU performed analysis of the microbiome. MD, LA, EF, MB, MM, and HZ recruited participants for the genetic studies. ER provided input on experimental design. BAJ was involved in patient recruitment, generation of EBV-immortalized cells, and data analysis. KK analyzed and interpreted the data. SSC, identified and enrolled the first family, the gene mapping, analyzed, and interpreted the data. VDD scored the histology slides, interpreted the data, and wrote the manuscript. JN conceived the experiments, analyzed and interpreted the data, wrote the manuscript, and obtained funding. AGG conceived the experiments, analyzed and interpreted the data, wrote the manuscript, and obtained funding.

## Supplementary Material

Supplemental data

Supporting data values

## Figures and Tables

**Table 2:**
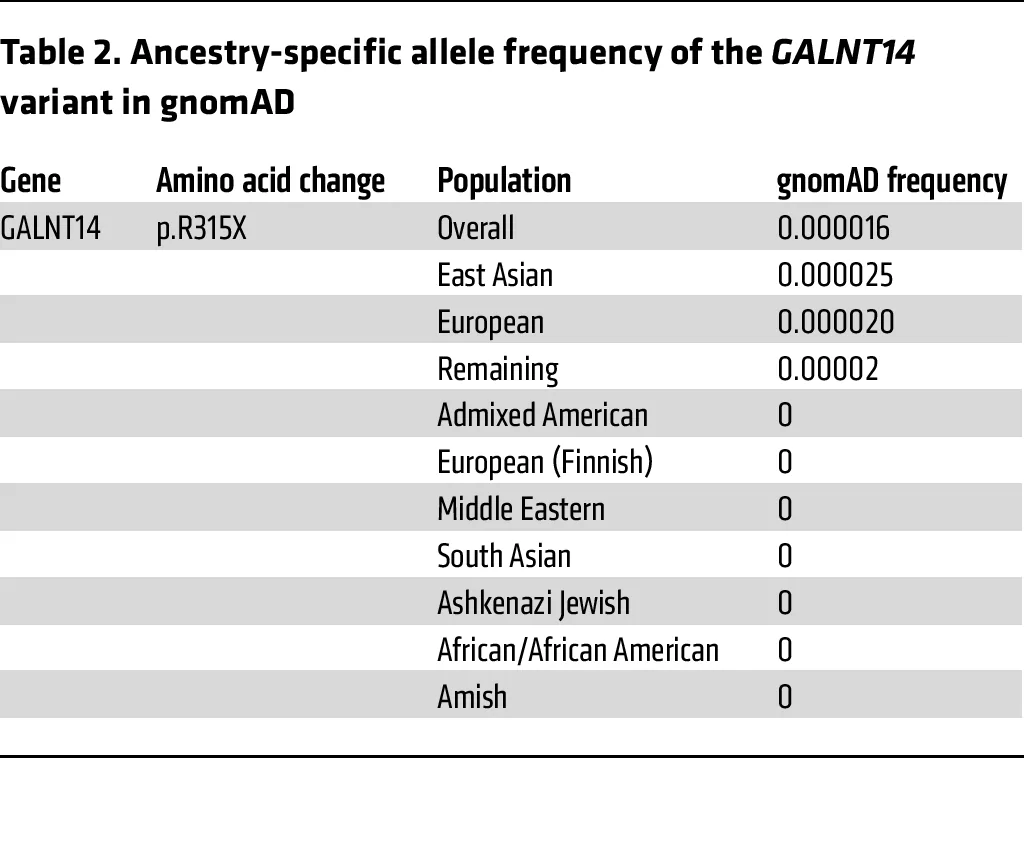
Ancestry-specific allele frequency of the *GALNT14* variant in gnomAD

**Table 1:**
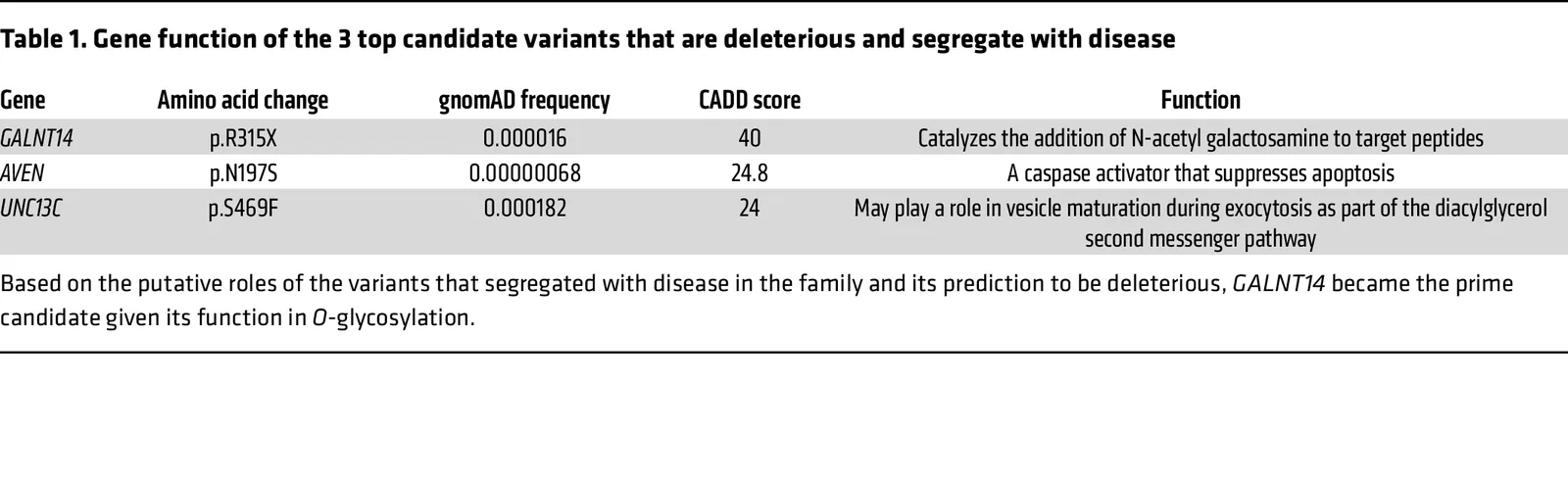
Gene function of the 3 top candidate variants that are deleterious and segregate with disease

## Version 1 03/28/2025

In-Press Preview

## Version 2 05/15/2025

Electronic publication

## Version 3 06/15/2026

Corrected an error in Figure 7

## Version 4 11/14/2026

Correction to figures 1M and 3E and supporting data values file.
