# Erratum to Loss of GalNAc-T14 links *O*-glycosylation defects to alterations in B cell homing in IgA nephropathy

**DOI:** 10.1172/JCI208844

**Published:** 2026-06-15

**Authors:** Sindhuri Prakash, Nicholas J. Steers, Yifu Li, Elena Sanchez-Rodriguez, Miguel Verbitsky, Isabel Robbins, Jenna Simpson, Sharvari Pathak, Milan Raska, Colin Reily, Anna Ng, Judy Liang, Natalia DeMaria, Amanda Katiraei, Kelsey O. Stevens, Clara Fischman, Samantha Shapiro, Swetha Kodali, Jason McCutchan, Heekuk Park, Djamila Eliby, Marco Delsante, Landino Allegri, Enrico Fiaccadori, Monica Bodria, Maddalena Marasa, Elizabeth Raveche, Bruce A. Julian, Anne-Catrin Uhlemann, Krzysztof Kiryluk, Hong Zhang, Vivette D. D’Agati, Simone Sanna-Cherchi, Jan Novak, Ali G. Gharavi

Original citation: *J Clin Invest*. 2025;135(10):e181164. https://doi.org/10.1172/JCI181164

Citation for this erratum: *J Clin Invest*. 2026;136(12):e208844. https://doi.org/10.1172/JCI208844

During the preparation of this manuscript, the *y* axis in Figure 7C was mislabeled. The correct figure is shown below, and the HTML and PDF versions of the article have been updated online.

The *JCI* regrets the error.

## Figures and Tables

**Figure 7:**
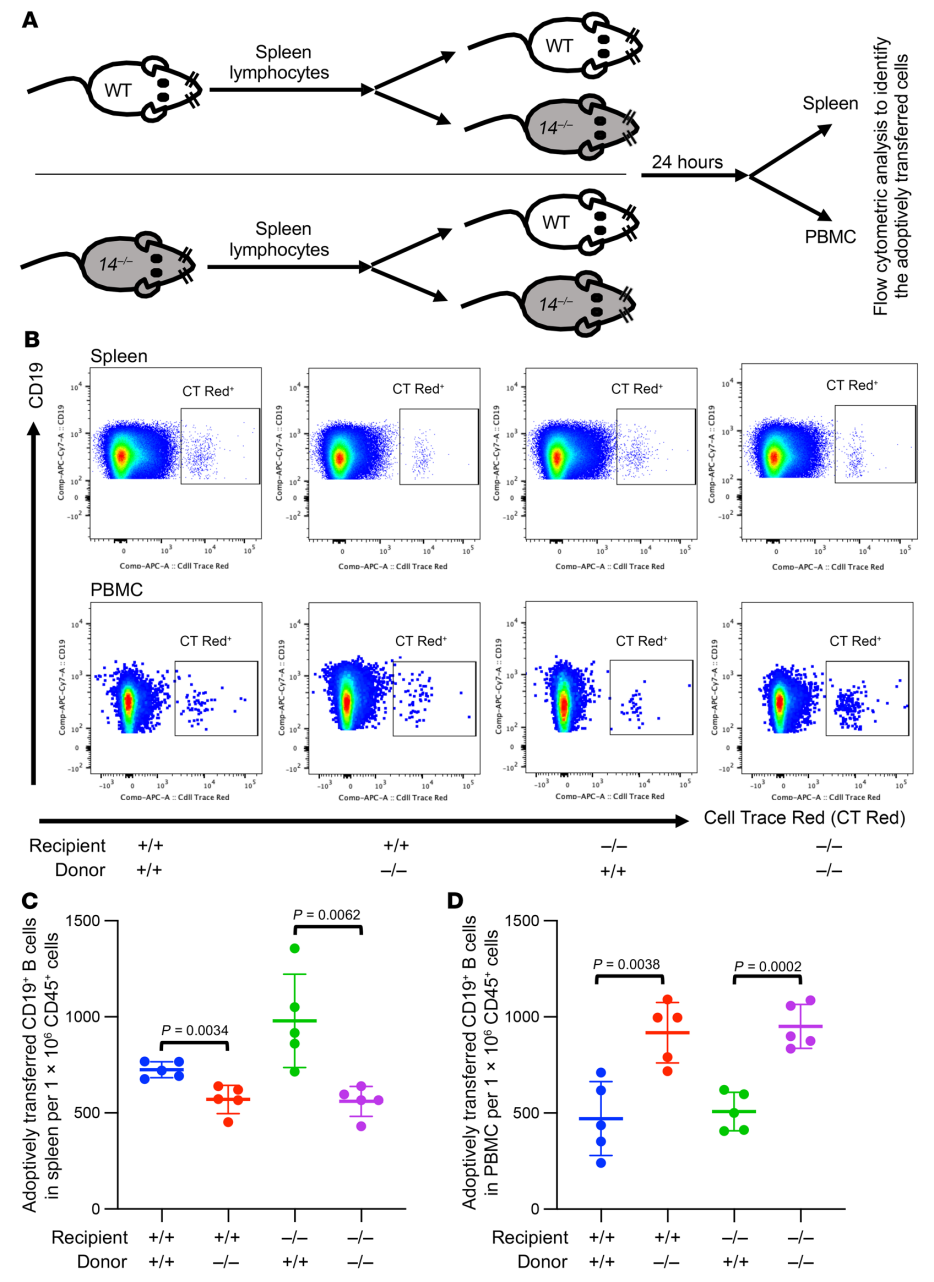
Adoptive transfer of lymphocytes from Galnt14^–/–^ mice demonstrates a deficiency in the homing ability of B cells.

## Version 1 06/15/2026

Electronic publication

