# Corrigendum to Loss of GalNAc-T14 links *O*-glycosylation defects to alterations in B cell homing in IgA nephropathy

**DOI:** 10.1172/JCI214000

**Published:** 2026-11-16

**Authors:** Sindhuri Prakash, Nicholas J. Steers, Yifu Li, Elena Sanchez-Rodriguez, Miguel Verbitsky, Isabel Robbins, Jenna Simpson, Sharvari Pathak, Milan Raska, Colin Reily, Anna Ng, Judy Liang, Natalia DeMaria, Amanda Katiraei, Kelsey O. Stevens, Clara Fischman, Samantha Shapiro, Swetha Kodali, Jason McCutchan, Heekuk Park, Djamila Eliby, Marco Delsante, Landino Allegri, Enrico Fiaccadori, Monica Bodria, Maddalena Marasa, Elizabeth Raveche, Bruce A. Julian, Anne-Catrin Uhlemann, Krzysztof Kiryluk, Hong Zhang, Vivette D. D’Agati, Simone Sanna-Cherchi, Jan Novak, Ali G. Gharavi

Original citation: *J Clin Invest*. 2025;135(10):e181164. https://doi.org/10.1172/JCI181164

Citation for this corrigendum: *J Clin Invest*. 2026;136(22):e214000. https://doi.org/10.1172/JCI214000

Following the publication of this article, the authors identified errors in Figures 1M and 3E as well as errors in the supporting data values for 2A and 7C. For Figure 1M, a calculation error was made, and baseline levels were reported that exceeded the predefined background threshold of 40 cycles. The correct figure appears below. The conclusions of Figure 1M remain the same.

For Figure 3E, a copy-and-paste error was made in the calculation for 2 data points in the *Galnt14^+/+^* and *Galnt14^–/–^* groups. The correct figure is shown below, and the conclusions of Figure 3E remain the same. 

The data in Figures 2A and 7D were correct in the original article; however, there were errors in the supporting data values for these figures. Additional minor typographical errors were also identified in the Supporting Data Values file. The corrected Supporting Data Values file has been posted online.

The authors regret the errors.

## Figures and Tables

**Figure 3:**
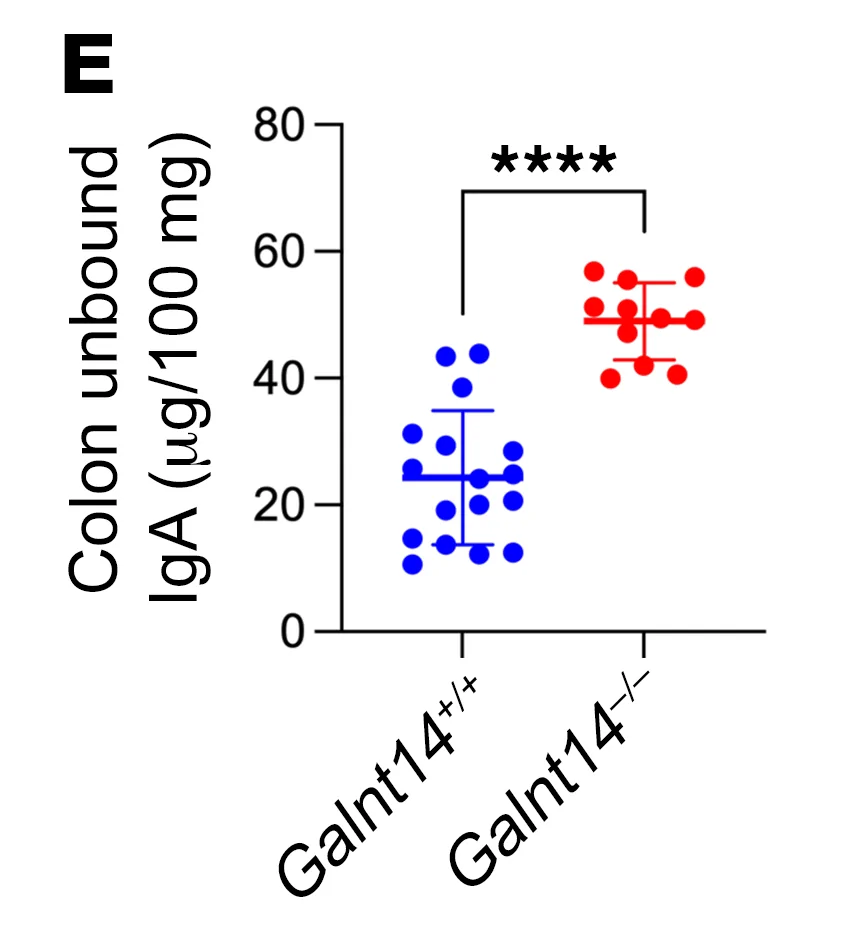


**Figure 1:**
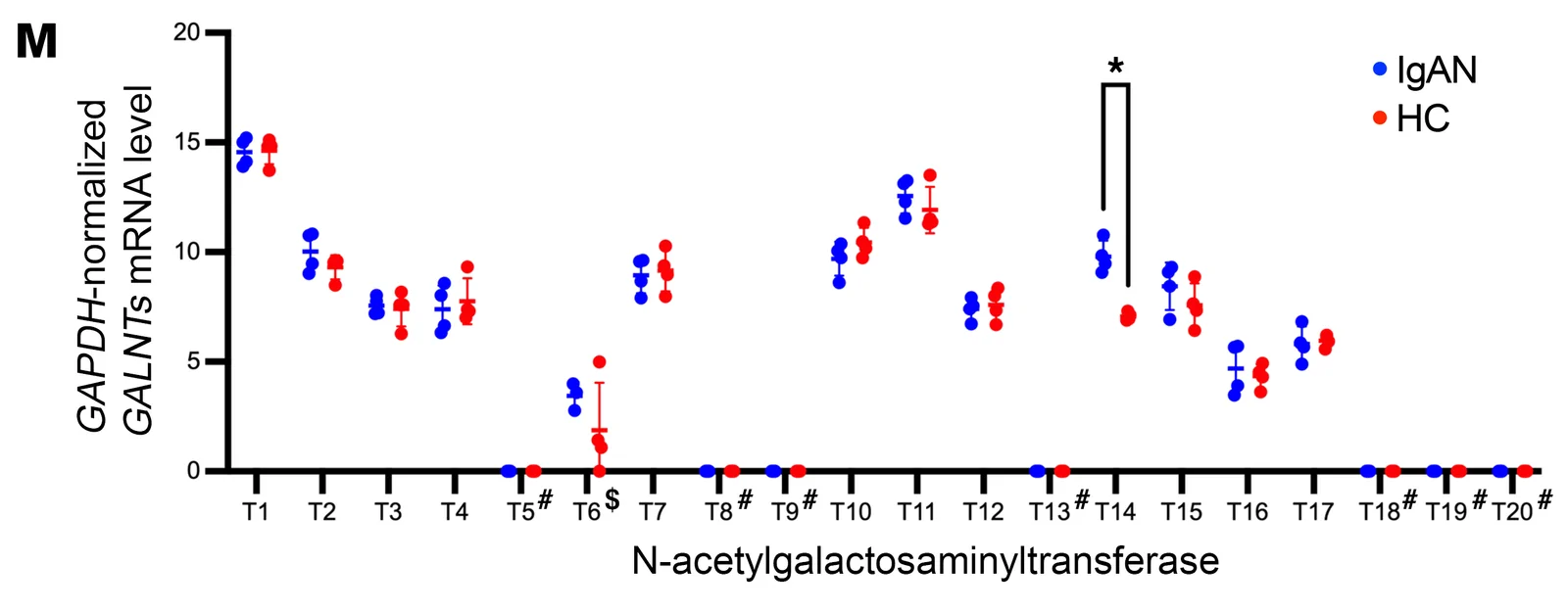
(**M**) Comparison of the expression of different *N*-acetylgalactosaminyltransferases in immortalized IgA1-secreting cell lines demonstrates elevated expression of *GALNT14* in the cells derived from the peripheral blood of patients with IgAN (*n* = 4) compared with those from healthy individuals (*n* = 4). *P = 0.006. ^#^Expression of theses genes could not be quantitated because the crossing point (Cp) exceeded the threshold for background level (40 cycles). ^$^One Cp value exceeded the assay threshold.

## Version 1 11/16/2026

Electronic publication

