# Supplementary material for: Loss of GalNAc-T14 links O-glycosylation defects to alterations in B cell homing in IgA nephropathy
Source: J Clin Invest. 2025 Mar 18;135(10):e181164. doi: 10.1172/JCI181164 (PMC12077892; doi:10.1172/JCI181164)
Supplement: Supplemental data [file jci-135-181164-s010.pdf]

Supplemental Figure 1.

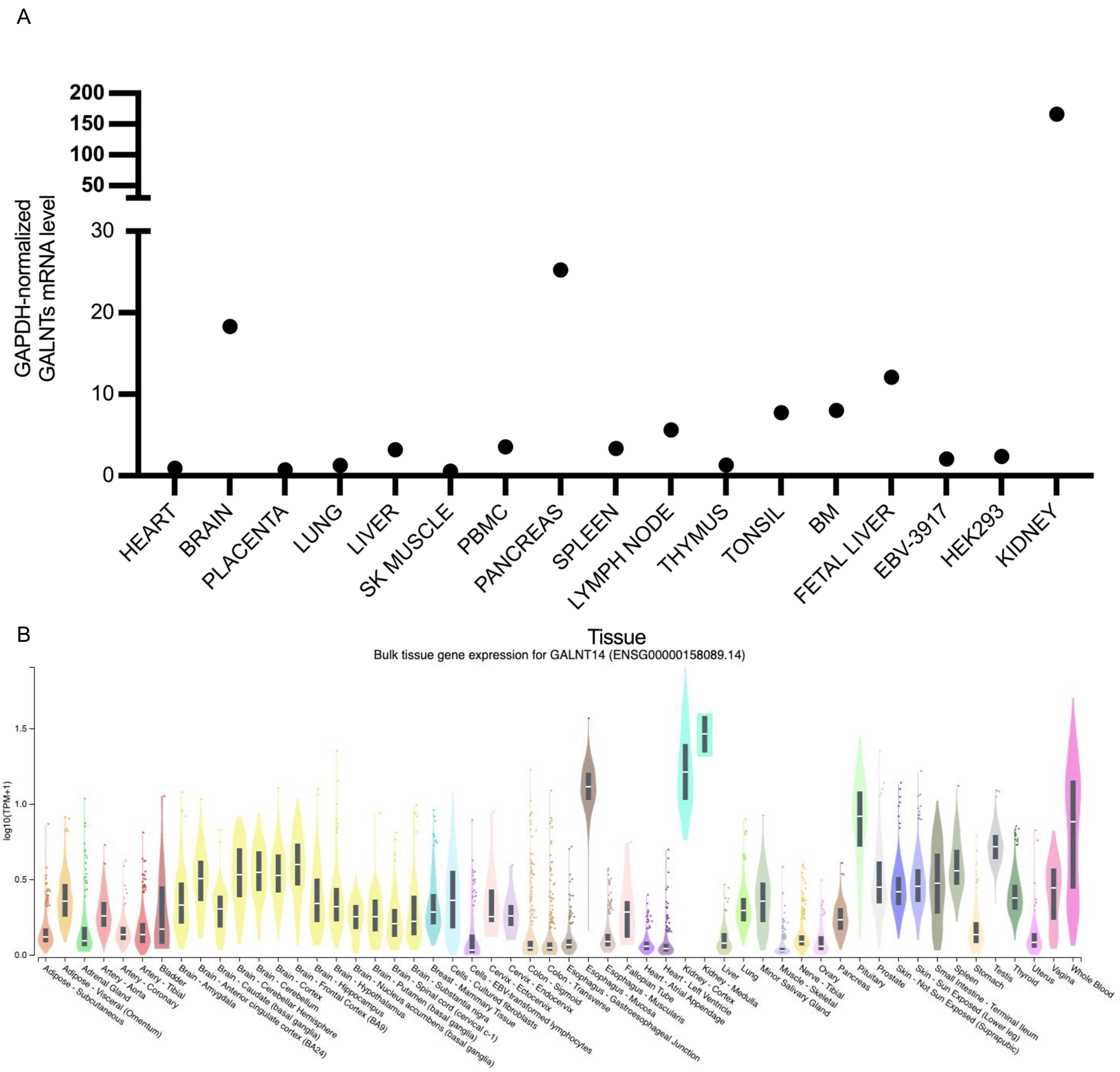

Supplemental Figure 1: Expression of *GALNT14* in human tissues. (A) Using a commercially available human cDNA library (Multiple Tissue panel and Immune system panel by Clontech), expression of *GALNT14* was highest in the human kidney. **(B)** *GALNT14* gene expression in tissue from the GTEx database again demonstrating *GALNT14* is highly expressed in the kidney.

Supplemental Figure 2.

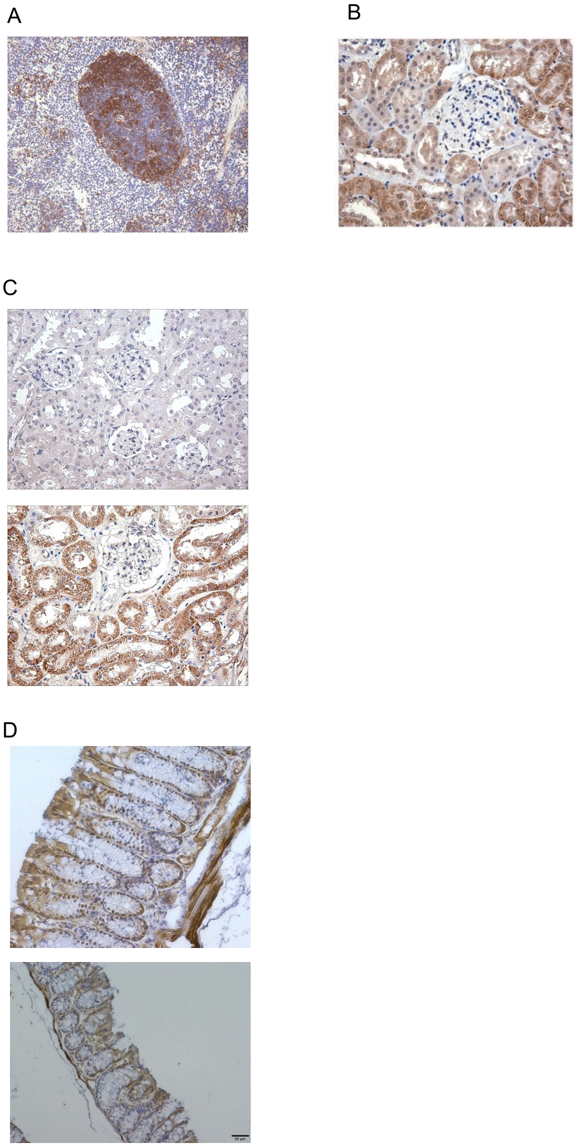

Supplemental Figure 2: Expression of GalNAc-T14 in mouse tissues: (A) Protein immunostaining of GalNAc-T14 in the mouse spleen is predominantly localized to the GC. **(B)** GalNAc-T14 staining of C57BL/6J mouse kidney demonstrated that Galnt14 is localized in the proximal and distal tubules. **(C)** Absence of GalNAc-T14 protein staining in the kidneys of *Galnt14*-null mice compared to the wild-type littermate control mice (n = 3 per genotype) **(D)** GalNAc-T14 staining of C57BL/6J mouse colon demonstrated localization in the goblet cells.

Supplemental Figure 3.

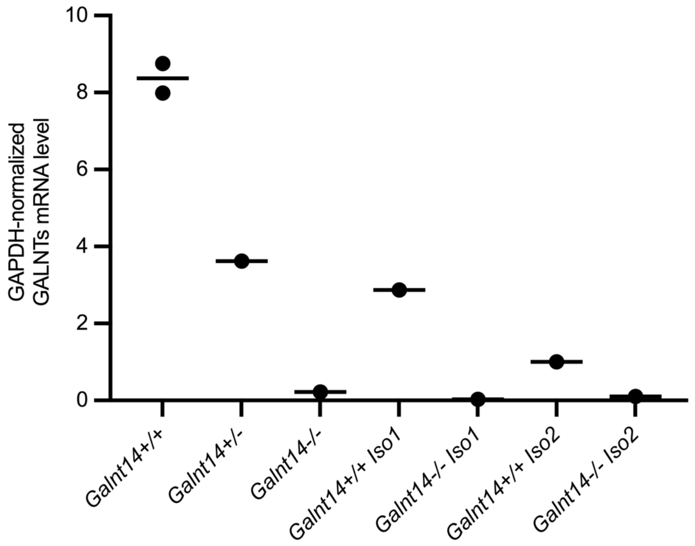

Supplemental Figure 3: Characterization of *Galnt14*-null mice. Semi-quantitative PCR of *Galnt14* expression in mouse kidney from wild-type, heterozygous, and *Galnt14*-null mice. Primer sequences available in Supplementary Table 2.

Supplemental Figure 4.

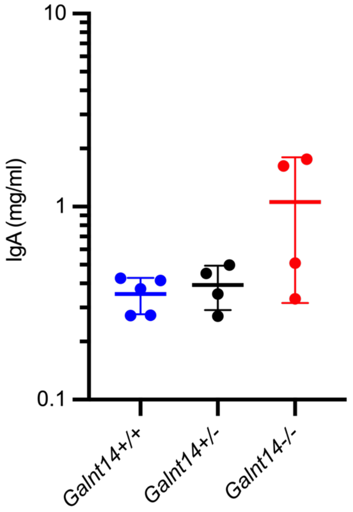

Supplemental Figure 4: IgA concentrations in the serum of *Galnt14-/-, Galnt14+/-* and *Galnt14+/+* mice. No difference of the IgA levels between the *Galnt14+/+* and *Galnt14+/-* mice. *Galnt14-/-* (n = 4), *Galnt14+/-* (n = 4), and *Galnt14+/+* mice (n = 5).

Supplemental Figure 5.

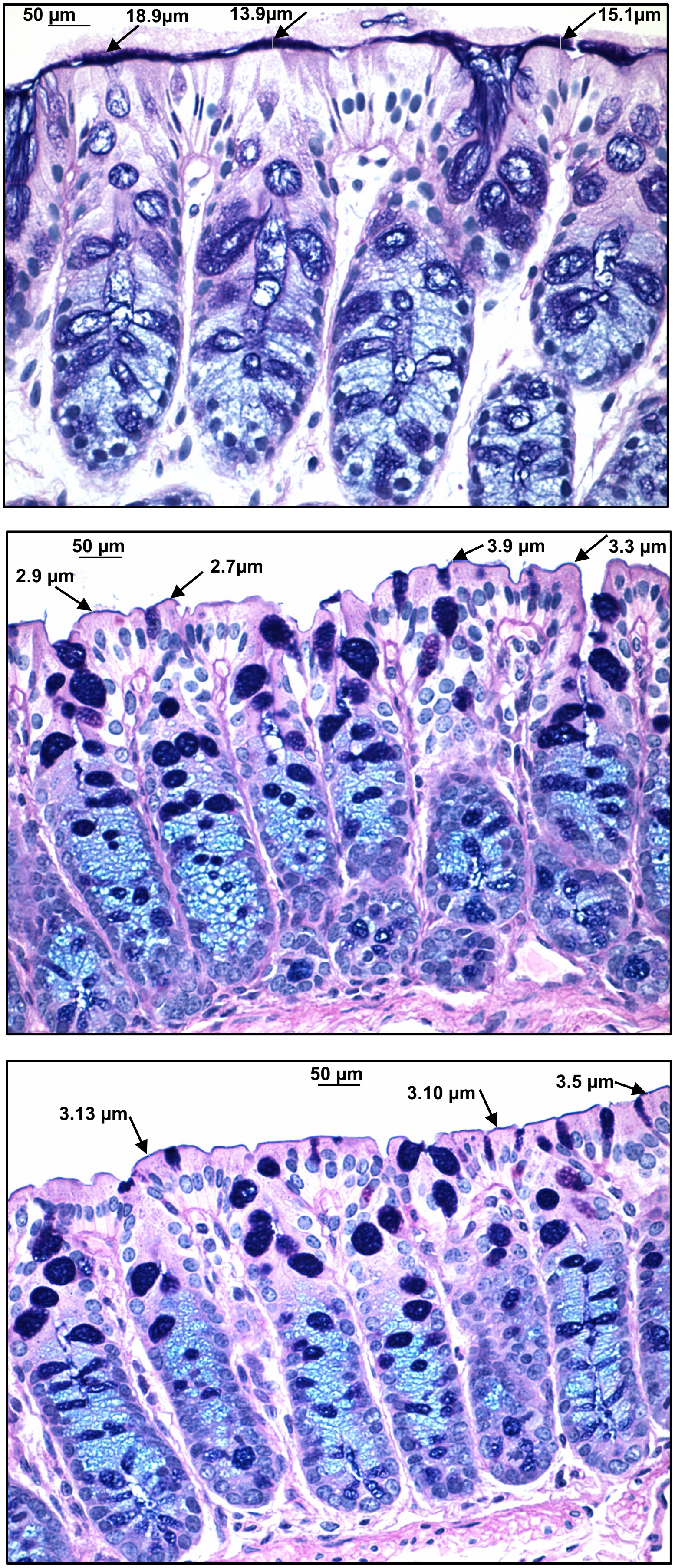

***Galnt14+/+* mice** — **Figure 2B bottom left panel**

***Galnt14-/-* mice** — **Figure 2B bottom middle panel**

***Galnt14-/-* mice** — **Figure 2B bottom right panel**

Supplemental Figure 5: Histological analysis of the colon reveals the mucin levels are significantly reduced in the *Galnt14-/-* mice compared to the *Galnt14+/+* mice. Images are at 600x magnification and enlarged from Figure 2B bottom panel with measurements of mucin thickness.

Supplemental Figure 6.

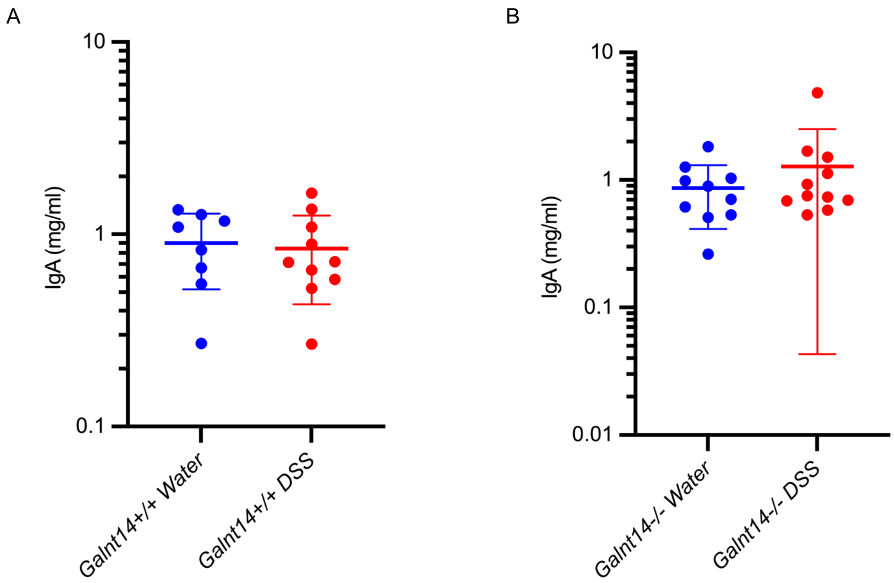

Supplemental Figure 6: IgA concentrations in the serum of *Galnt14-/-* and *Galnt14+/+* mice treated with water or 2.5% DSS in water. (A) No difference of the IgA levels in *Galnt14+/+* mice treated with water only (n = 8, 5♂ and 3♀) or 2.5% DSS in water (n =10, 7♂ and 3♀). **(B)** No difference of the IgA levels in Galnt14-/- mice treated with water only (n = 10, 4♂ and 6♀) or 2.5% DSS in water (n = 11, 6♂ and 5♀).

Supplemental Figure 7.

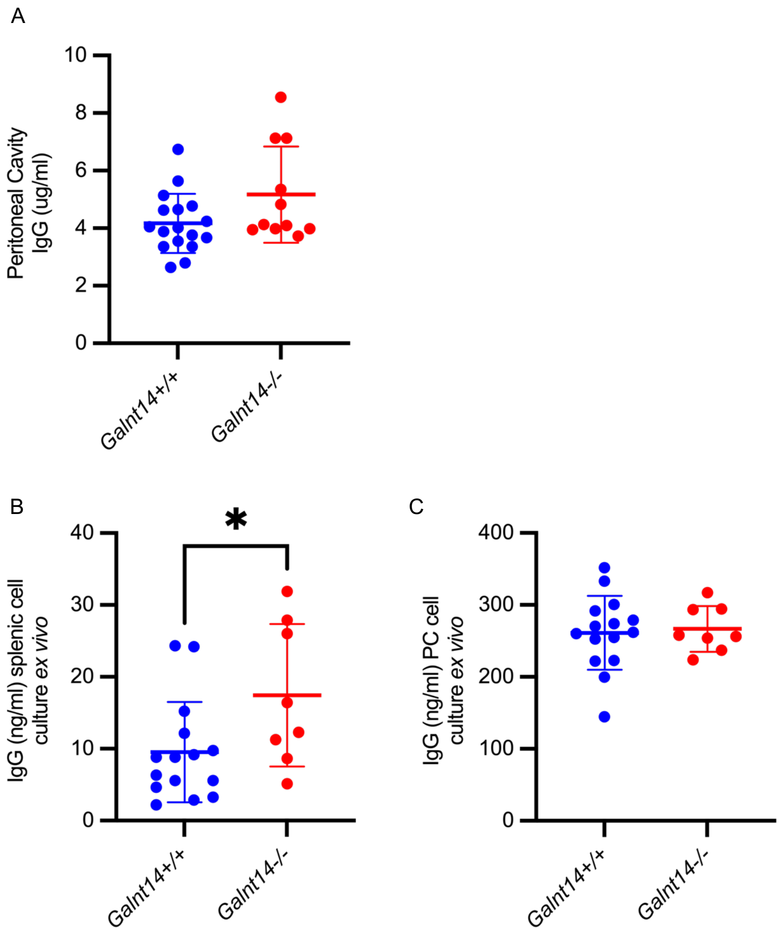

Supplemental Figure 7: IgG concentrations in the mucosal compartments of *Galnt14-/-* and *Galnt14+/+* mice. (A) No difference of the IgG levels in the peritoneal cavity was detected between *Galnt14-/-* (n = 11, 6♂ and 5♀) and *Galnt14+/+* mice (n = 17, 10♂ and 7♀). **(B)** Cultured splenic lymphocytes from *Galnt14-/-* mice (n = 8, 4♂ and 4♀) secreted more IgG ($P < 0.05$) than those from *Galnt14+/+* mice (n = 15, 9♂ and 6♀). **(C)** No difference was observed in IgG production by cultured peritoneal-cavity lymphocytes between *Galnt14-/-* (n = 8, 4♂ and 4♀) and *Galnt14+/+* mice (n = 15, 9♂ and 6♀).

Supplemental Figure 8.

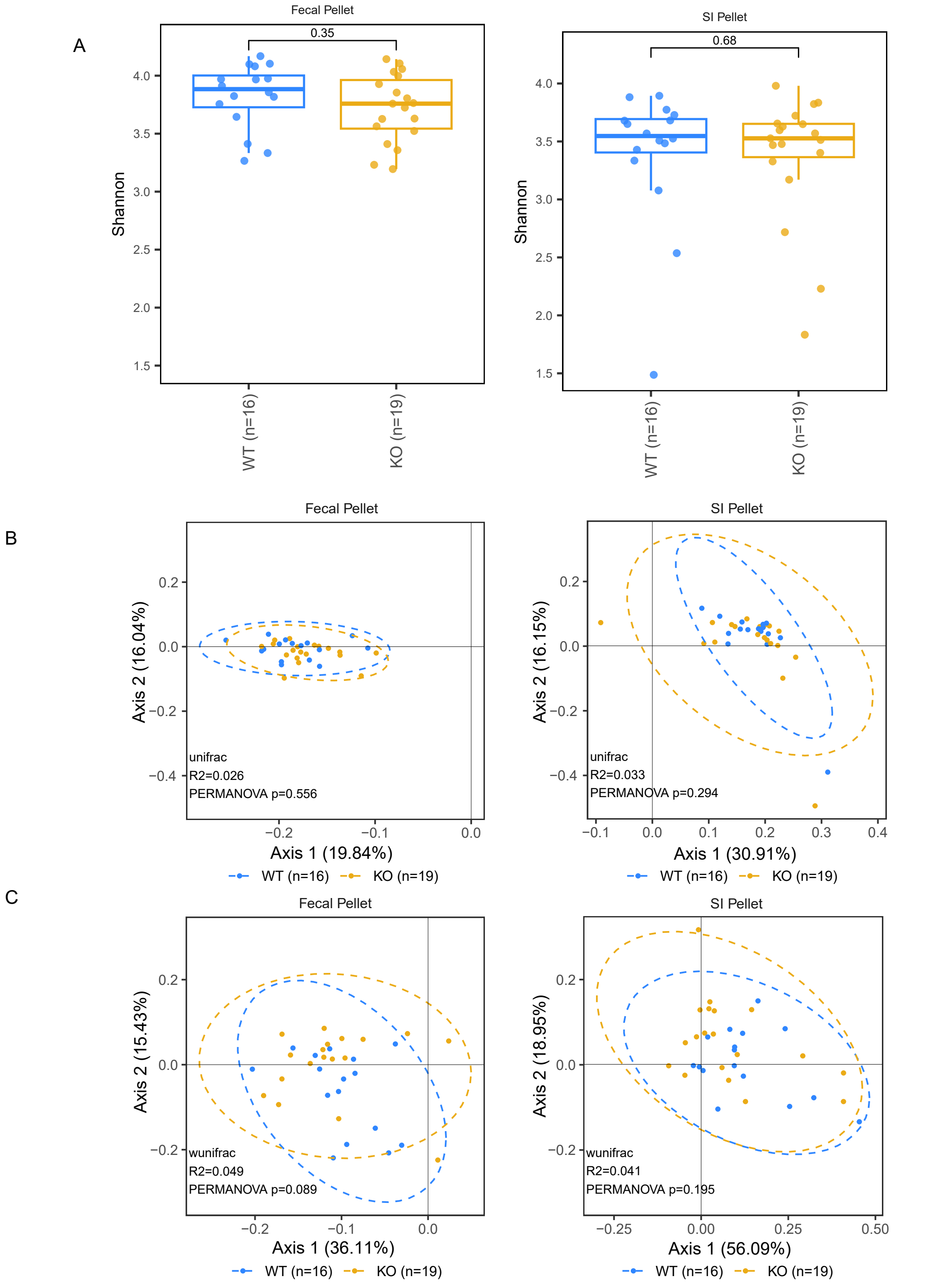

Supplemental Figure 8: Microbiome analysis of the fecal pellets and the small intestine of *Galnt14*+/+ mice and *Galnt14-/-* mice. (A) Alpha diversity comparisons (Shannon Index) of the fecal pellets and the small intestine. **(B)** Principal Coordinates Analysis (PCoA) of microbial community composition across *Galnt14+/+* mice and *Galnt14-/-* mice, of the Fecal and Small Intestine (SI) Pellets using Unifrac (Beta diversity). **(C)** Principal Coordinates Analysis (PCoA) of microbial community composition across *Galnt14+/+* mice and *Galnt14-/-* mice, of the Fecal and Small Intestine (SI) Pellets using Wunifrac (Beta diversity).

Supplemental Figure 9.

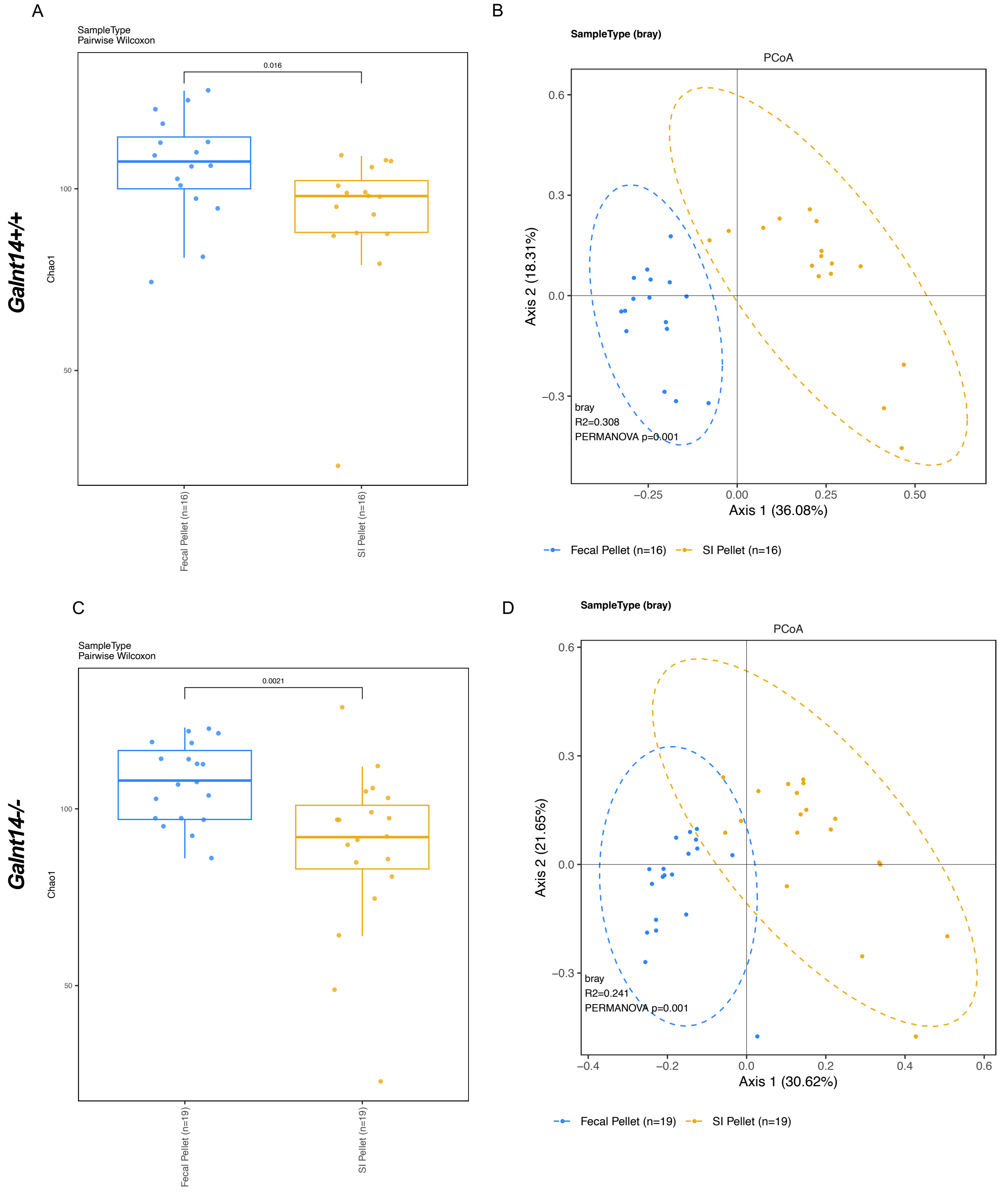

Supplemental Figure 9: Microbiome analysis comparing fecal pellets and the small intestine of *Galnt14+/+* mice and *Galnt14-/-* mice. (A) Alpha diversity comparisons (Chao1) between the fecal pellets and the small intestine of *Galnt14+/+* mice (n = 16 9♂ and 7♀): note, robust linear regression to account for the influence of sex indicated significant differences remained: P = 0.016 **(B)** Beta diversity (Bray) diversity comparisons between the fecal pellets and the small intestine of *Galnt14* mice. **(C)** Alpha diversity comparisons (Chao1) between the fecal pellets and the small intestine of *Galnt14-/-* mice (n = 19, 12♂ and 7♀); note, robust linear regression to account for the influence of sex indicated significant differences remained: P = 0.002. **(D)** Beta diversity (Bray) diversity comparisons between the fecal pellets and the small intestine of *Galnt14-/-* mice.

Supplemental Figure 10

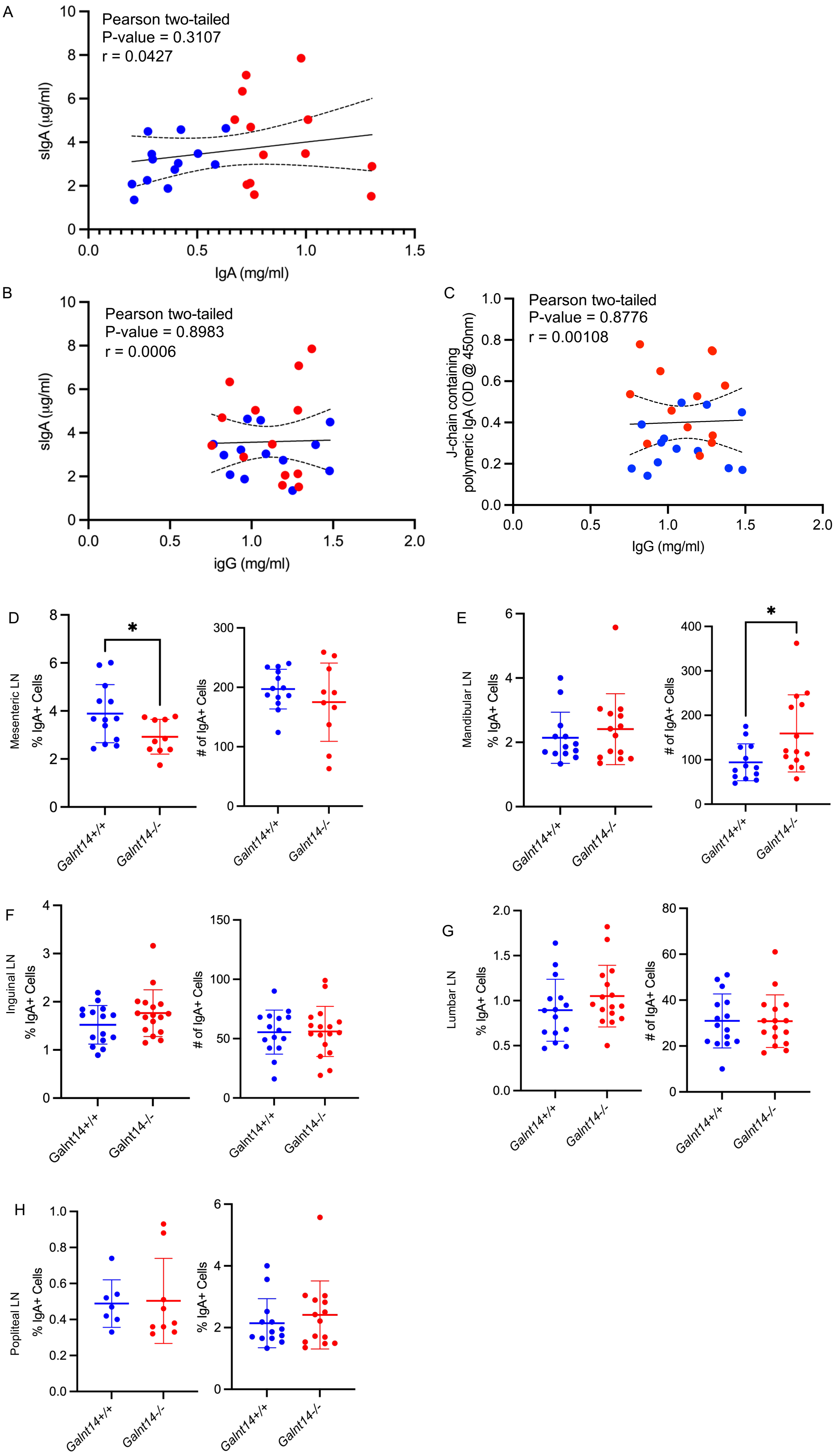

Supplemental Figure 10: sIgA1 and J-Chain analysis in *Galnt14+/+* and *Galnt14-/-* mice. (A) No correlation between serum levels of sIgA and IgA (*Galnt14-/-* mice (n = 13, 6♂ and 7♀) and *Galnt14+/+* mice (n = 13, 7♂ and 6♀), (P = 0.3107, Pearson two-tailed test). **(B)** No correlation between serum levels of sIgA and IgG (*Galnt14-/-* mice (n = 13, 6♂ and 7♀) and *Galnt14+/+* mice (n = 13, 7♂ and 6♀), (P = 0.8983, Pearson two-tailed test). **(C)** No correlation between serum levels of polymeric IgA (i.e., IgA with J-Chain) and serum IgG (*Galnt14-/-* mice (n = 13, 6♂ and 7♀) and *Galnt14+/+* mice (n = 13, 7♂ and 6♀), (P = 0.8776, Pearson two-tailed test). **(D)** A significant decrease in the percentage but not the number of IgA+ B cells in the mesenteric lymph node of *Galnt14-/-* mice (n = 10, 5♂ and 5♀) compared to *Galnt14+/+* mice (n = 13, 6♂ and 7♀). **(E)** A significant increase in the number but not the percentage of IgA+ B cells in the mandibular lymph node of *Galnt14-/-* mice (n = 14, 7♂ and 7♀) compared to *Galnt14+/+* mice (n = 13, 7♂ and 6♀). **(F)** No differences in the percentage and the number of IgA+ B cells in the inguinal lymph node of *Galnt14-/-* mice (n = 17, 9♂ and 8♀) compared to *Galnt14+/+* mice (n = 15, 8♂ and 7♀). **(G)** No differences in the percentage and the number of IgA+ B cells in the lumbar lymph node of *Galnt14-/-* mice (n = 16, 8♂ and 8♀) compared to *Galnt14+/+* mice (n = 15, 8♂ and 7♀). **(H)** No differences in the percentage and the number of IgA+ B cells in the popliteal lymph node of *Galnt14-/-* mice (n = 9, 6♂ and 3♀) compared to *Galnt14+/+* mice (n = 7, 5♂ and 2♀).

Supplemental Figure 11.

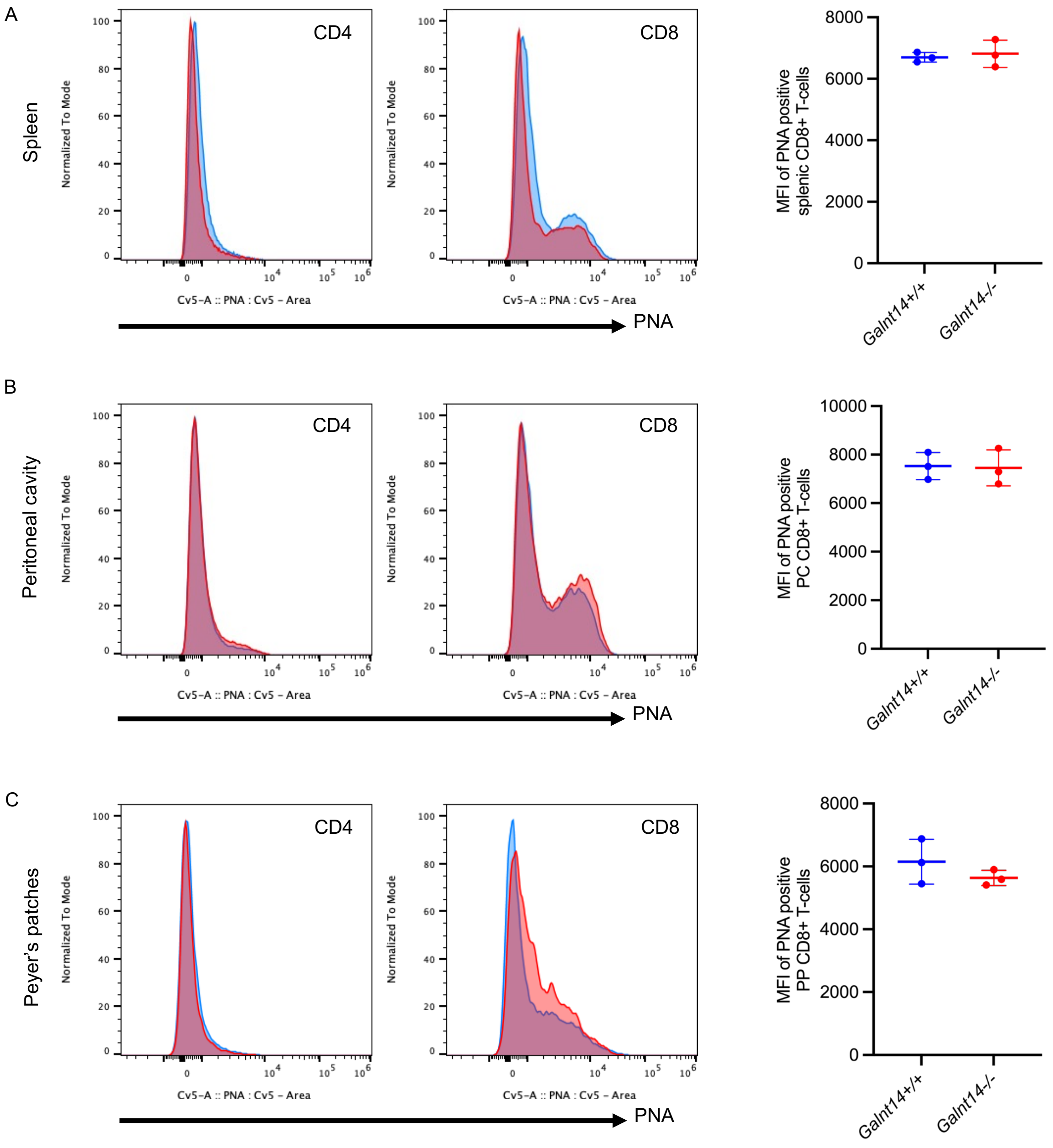

Supplemental Figure 11: PNA staining of T-cells in *Galnt14+/+* and *Galnt14-/-* mice. (A) Splenic CD4+ T-cells were negative for PNA staining (left panel) and a sub-population of CD8+ T-cells were PNA-positive measured by flow cytometry (middle panel). No differences in the MFI of the splenic PNA-positive CD8+ T cells were observed between the *Galnt14+/+* (n = 3♀) and *Ganlnt14-/-* mice (n = 3♀). **(B)** Peritoneal cavity CD4+ T-cells were negative for PNA staining (left panel), and a sub-population of CD8+ T-cells were PNA-positive measured by flow cytometry (middle panel). No differences in the MFI of the peritoneal cavity PNA-positive CD8+ T cells were observed between the *Galnt14+/+* and *Galnt14-/-* mice. **(C)** The Peyer's patches CD4+ T-cells were negative for PNA staining (left panel), and a sub-population of CD8+ T-cells were PNA-positive measured by flow cytometry (middle panel). No differences in the MFI of the Peyer's patches PNA-positive CD8+ T cells were observed between the *Galnt14+/+* and *Galnt14-/-* mice.

Supplemental Figure 12.

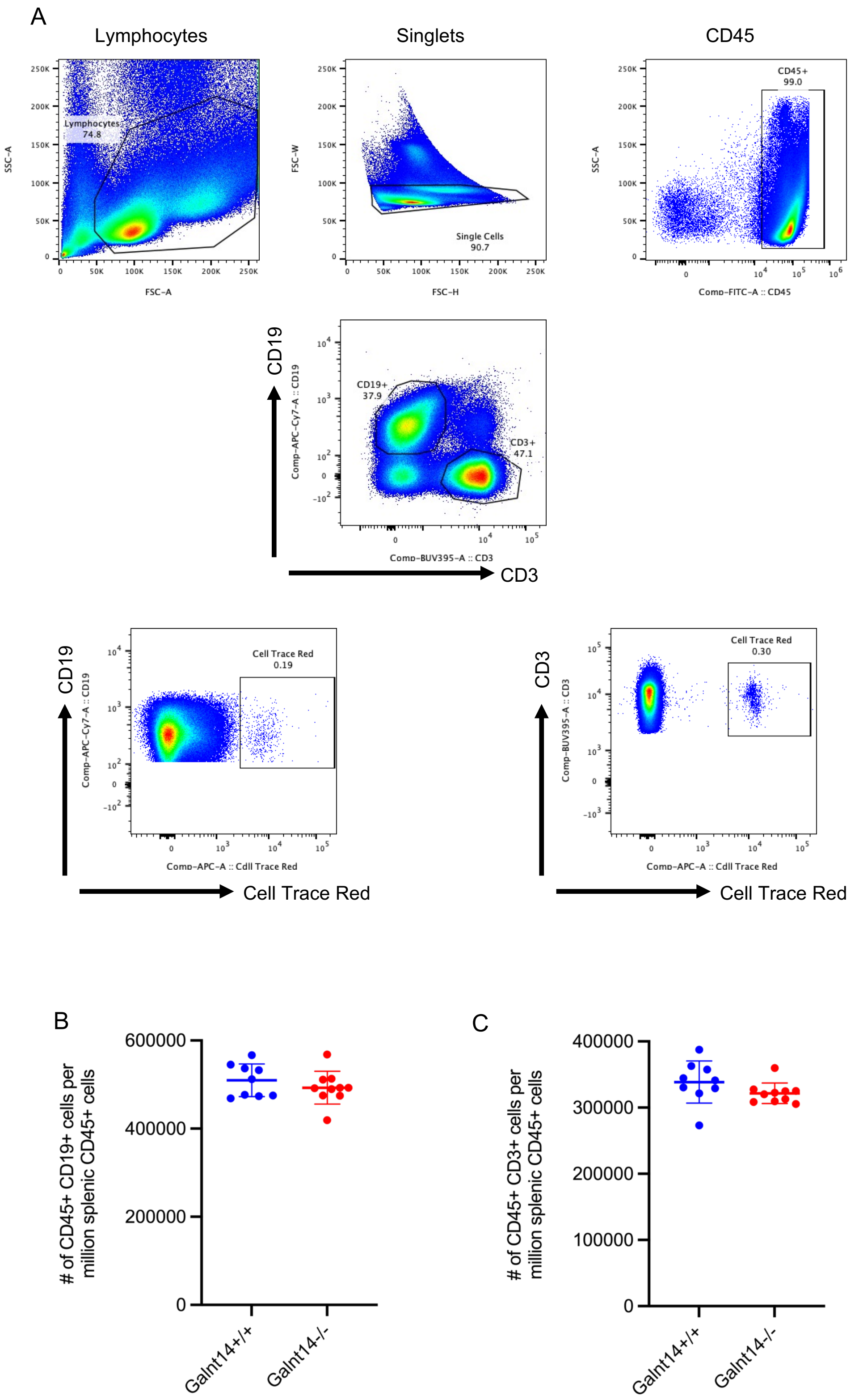

Supplemental Figure 12: Adoptive transfer experiments. (A) Identification of the adoptively transferred cells in the spleen and the PBMC. **(B)** Analysis of the CD19+ B cells derived from the spleens of *Galnt14*-/- (n = 10) and *Galnt14*+/+ (n = 9) mice (excluding the adoptively transferred cells) demonstrating no significant difference in the number of CD19+ B cells. **(C)** Analysis of the CD3+ T-cells derived from the spleens of *Galnt14*-/- (n = 10) and *Galnt14*+/+ (n = 9) mice (excluding the adoptively transferred cells) demonstrating no difference in the number of CD3+ T-cells.

Supplemental Figure 13.

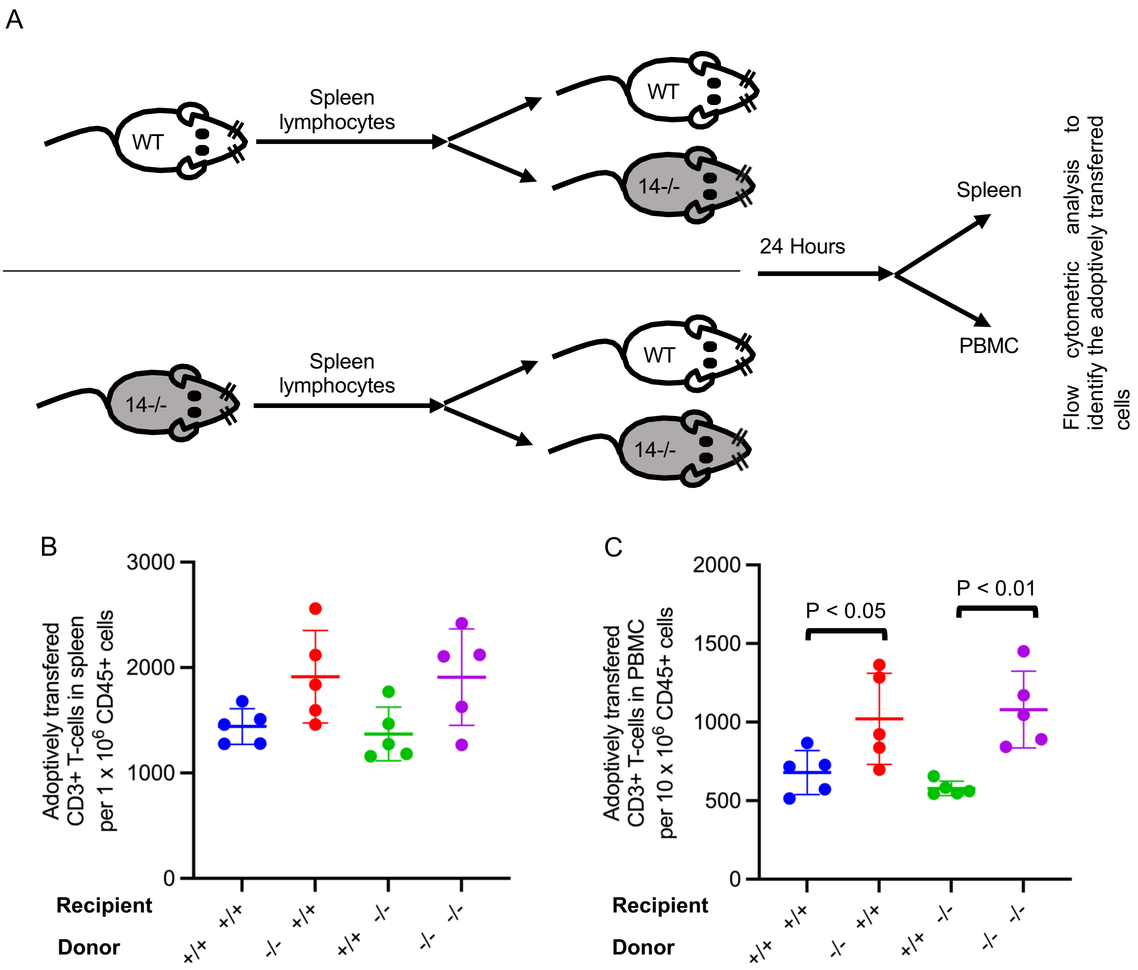

Supplemental Figure 13: Adoptive transfer experiments of CD3+ T-cells. (A) Schematic of the adoptive transfer experiment. **(B)** Adoptive transfer of 3.5 x 10^6 lymphocytes derived from *Galnt14-/-* mice had no differences in the adoptively transferred CD3+ T cells identified in the spleens of the recipient mice (*Galnt14+/+* or *Galnt14-/-*, n = 5 per group, 2♂ and 3♀) compared to adoptively transferred lymphocytes derived from *Galnt14+/+* mice into recipient mice (*Galnt14+/+* or *Galnt14-/-*, n = 5 per group). **(C)** Adoptive transfer of 3.5 x 10^6 lymphocytes derived from *Galnt14-/-* mice had increased ($P < 0.05$) CD3+ T cells identified in the PBMC of the recipient mice (*Galnt14+/+* or *Galnt14-/-*, n = 5 per group) compared to adoptively transferred lymphocytes derived from *Galnt14++* mice into the recipient mice (*Galnt14+/+* or *Galnt14-/-*, n = 5 per group, 2♂ and3 ♀).

Supplemental Table 1: Regions of genome-wide linkage and the variants that segregate with disease in the family.

Chr Band (GRCh37p.12 /hg 19)	Start (BP)	End (BP)	Start SNP	End SNP	Size (bp)	N. of Genes	Segregating Variants Predicted to be Damaging
1p36.33 - 1p36.32	944564	1521895	rs3128117	rs3128117	577331		
1p36.13-1p36.13	15431657	17753639	rs494983	rs6688886	2321982	102	
2p24.1-2p22.3	24358652	37827519	rs13035774	rs874427	13468867	178	GALNT14- p.R315X
9q22.31-9q31.1	92925179	101008885	rs7468504	rs7853442	8083706	143	
11q12.3-11q13.1	60181627	65399528	rs1443241	rs2009453	5217901	223	
15q13.1-15q22.2	33767621	68877435	rs1399073	rs1994714	35109814	490	AVEN - p.N197S UNC13C - p.S469F
21q21.3-21q22.3	32444209	47512330	rs1571691	rs2236490	15068121	275	
Total					79847722	1411	
% of Genome/Genes					2.46%	8.85%	

Genome-wide linkage analysis under the autosomal dominant model with incomplete penetrance revealed 7 regions in the genome that segregate with disease-harboring 1411 genes that comprise 2.5% of the genome. Variants that are predicted to be deleterious and segregate with disease in the family are tabulated in the respective genomic regions.

Supplemental Table 2: Filtering pipeline to find pathogenic variants in the IgAN family.

**Filtering Pipeline**	**# of variants**
Rare (MAF < 0.0001), high-quality variants in all chromosomes	1310
Variants shared in both cases in autosomes	898
Heterozygous variants LOD > 0	14
Heterozygous variants that are validated and segregate with phenotype	8
Heterozygous variants that are predicted to be deleterious	3
Variants that are independently enriched in IgAN cases	1

A strict filtering pipeline was employed on the variants that resulted from exome sequencing of the 2 individuals (1 case of IgAN and 1 individual with hematuria) in the pedigree. High-quality variants that passed strict quality-control filters including a qual score > 50 for insertion deletions and single nucleotide variants, read depth > 10, allelic depth > 0.25, and genotype quality depth > 20 that were rare (MAF < 0.0001) were filtered in. Of these, 898 variants were shared among the 2 individuals sequenced and were present on autosomes. A LOD score > 0 was applied to filter variants that are present in the shared genomic regions in the family that were then validated using Sanger Sequencing leaving 8 variants. Three variants were predicted to be deleterious based on CADD. Only 1 LOF variant in *GALNT14* remained.

Supplemental table 3: Colonic injury score following treatment of mice with water or DSS.

**Genotype**	**Condition**	**Number of Mice**	**Average Score**	**Standard Deviation**
Galnt14-/-	Water	8	0.25	0.46
Galnt14-/-	DSS	8	6.625	0.85
Galnt14+/-	Water	5	0.4	0.54
Galnt14+/-	DSS	4	6.75	1.,25
Galnt14+/+	Water	6	0	0
Galnt14+/+	DSS	8	6.625	1.68

A composite injury score of the colon was calculated for each mouse using histological criteria which includes % erosion of the crypt, % of inflammatory cells, apoptosis, necrosis, epithelial regeneration, and crypt abscess following treatment.

Supplemental Table 4: Percent of bacteria that high/positively stained for IgA determined by flow cytometry.

Mouse	Wild Type	*Galnt14*-/-
Av	52.51%	69.56%
SD	6.97	8.049
t-test		0.00175

Supplemental Table 5: Primers for Sanger validation and RealTime PCR of *GALNT14*

Forward Primer	Reverse Primer	Size (BP)
T14-exon10- TAA CAC TGG ATG TGA CAG GC	T14-exon10-GTT AGA AGG CAA CTC TCT TAG TG	662
T14-exon1-CCC GAG CGT TTT AAA ATC CAG	T14-exon1-CCT TCC CTG CCA CGT TT	676
T14-exon2-CTT TGA GCC AAA CCT TGC C	T14-exon2-ACA GCC AGG GTC AAT CAC	455
T14-exon3-CC CTT CTA GAT ATC AAG CAT CTG	T14-exon3-GGA GTG ACT GAG TGG ACA C	527
T14-exon4-CTC CTG GCT CAC AGA TAA GC	T14-exon4-TTA CAG AGT GGC TGG GAA C	284
T14-exon5-TGC ACT CTG CAG TTA GGG	T14-exon5-GAG AAG CCA CCC AGC ATT	306
T14-exon6-TCG CTG GAC AAT GGA GTG	T14-exon6-TTC CTT CAT CAA TAA AAT CGG GAC	327
T14-exon7-GCA TCA GAA CTG GAG AGG TC	T14-exon7-CCA TCC ACA GTC CTG GTG	453
T14-exon8-AGA GGT GGT AGT GGG TAG AG	T14-exon8-CCA TCC CCT TAG CAA GAA GT	495
T14-exon9-CAA GGT CAG GAG ATT TAC AAT CAC	T14-exon9-TCT CTG ACA GCT TGG TCT TG	454
T14-exon11-GGA ATA GGA AAG AAG CCA CAG	T14-exon11-CAA CCT GTA CTA AAA TCT TGT GTC	620
T14-exon12-AGA TAT TGC TAA GTG TTT CCA TAG C	T14-exon12-CTG GCT AAC TGT AAT CCT TCA TG	629
T14-exon13-CCC TGA CTT ACA GAC TCA CTG	T14-exon13-GGA TGC TGC CTA TTA AAC TGT C	612
T14-exon14-AGC CCA GAA TTG TTC AGG AC	T14-exon14-TTT CAT GGG GCA GGC TG	785
T14-exon15-CAT GAG ACC TGC CTC TCT G	T14-exon15-CTC CCT GAG ACA GTT GCT C	474
T14Isoform2-exon2-GGT GAA GTG TAG TCT GTT TTC TC	T14Isoform2-exon2-AAA CCA GGT CTT CTA CTA GCA C	498
T14Isoform2-exon3-ATA AGA ATG TTC AGC TTA TAA AAA TTA GTG	T14Isoform2-exon3-AAG GAT GGT TGC TGT AGA GAC	1819
cDNA_F-GAT ATC ATT AAC CTG GAC ACC TTC	cDNA_R-CCA GAT GTC CAT GTC CAT ATC ATA	NA

Supplemental Table 6. Primers specific for *GALNT* genes and a housekeeping gene (*GAPDH*) used for RealTime PCR

Gene	Primer orientation	Sequence
GALNT1	Forward	CGCTCACCAAGACACATGAT
GALNT1	Reverse	GCCATCCCACTGTACACTCA
GALNT2	Forward	GAACAGATCGAGGGCAACTC
GALNT2	Reverse	TCGCGGAAAGTTTAAGAAACA
GALNT3	Forward	AATCATTTTGGTGGATGATGC
GALNT3	Reverse	CGACAGCCGTGTAGTTCTCA
GALNT4	Forward	TCTGTCCCCAAACAGGAAAG
GALNT4	Reverse	TTTTCACCTCCCCACACTTC
GALNT5	Forward	CTGGATGTGCAGAGCAGCTA
GALNT5	Reverse	TGGACAGGCCACTTTCTTTC
GALNT6	Forward	CCTGAGTGTGTGGACCAGAA
GALNT6	Reverse	CAGCTGCTTCACGTACTGCT
GALNT7	Forward	CACTTCGAGCGTTGTCATTG
GALNT7	Reverse	GCACCAATACTTCGTGCTTG
GALNT8	Forward	GGATGAGCACAAACACATGG
GALNT8	Reverse	CCCTGATCCAAGCAGACATT
GALNT9	Forward	TGCCTGACTCCAAGTGTCTG
GALNT9	Reverse	TGCGTGTTTGATCCAGTTTC
GALNT10	Forward	GTATGACCCAGGCTTGGAGA
GALNT10	Reverse	TCTGCGTACTCATCCATCCA
GALNT11	Forward	CCCCCTTTCTGAGCTAGGAC
GALNT11	Reverse	TCCATATGGTCGCCTTTTTC
GALNT12	Forward	TGGCACACAGTTCCTGAGAG
GALNT12	Reverse	AGCTTGCTTGGGGAAAACAT
GALNT13	Forward	GAAGCTTGGAGCACTCTCCTT
GALNT13	Reverse	GGCACGTATTAACCCAGAGC
GALNT14	Forward	TATTAAACCGCACCCCTACG
GALNT14	Reverse	CCAGTCCCTGTTCACCTCAC
GALNT15	Forward	GGACTTTGGGGAATCCCAGG
GALNT15	Reverse	TTGAGTTGTCCTTGCTGGCT
GALNT16	Forward	ACTGCAGATGTGCAACCCTA
GALNT16	Reverse	ATGTGAGGAGATGGGAACAGC
GALNT17	Forward	GGAACCAGCTCTGGGGATAC
GALNT17	Reverse	TCTAGGAGTTGGCATGGTGG
GALNT18	Forward	GGGTGCCGTAACCTCTCATT
GALNT18	Reverse	TTGATGCGGGTGAGTACAGG
GALNT19	Forward	CCTTGGGCTCATTGAAGGTT
GALNT19	Reverse	GCGTTTGTGGACATACTCCT
GALNT20	Forward	AATCCGGTCACCTGCAATGT
GALNT20	Reverse	CACGTAATTCCACGATGTTCCA

GAPDH	Forward	GAGTCAACGGATTTGGTCGT
GAPDH	Reverse	TTGATTTTGGAGGGATCTCG

Supplemental Table 7: Primers for *Galnt14* genotyping of mice.

Forward Primer	Reverse Primer	Size
T14-WT CCT CGG AGT CAG TCT GTC TGT C	T14-WT GCA GTA CGG TGA TCC AGA GCA C	500
T14-KO GCA GCG CAT CGC CTT CTA TCG CC	T14-KO CTG CAT TTG AAC CCT CAA TCC CAG	250

Methods

Recruited Families and Selection of Sequencing Cohort. The study protocol was approved by the Institutional Review Board at Columbia University Irving Medical Center and collaborating medical centers and signed written informed consent by the participants was obtained. Ten well-characterized pedigrees with multiple individuals affected with IgAN (at least two biopsy-proven cases/family) were selected for exome sequencing. Families exhibited autosomal dominant inheritance with incomplete penetrance. All individuals with IgAN and an individual with hematuria from each family were selected for exome sequencing. The coding sequence of *GALNT14* was confirmed by the Sanger method in an additional familial IgAN cohort comprising of 68 cases of European and Asian ancestry. Selected variants of *GALNT14* were sequenced in a large control population depending on where the patient with the variant originated. In total, this cohort consisted of 308 individuals from Italy.

DNA Extraction. DNA was extracted from peripheral blood from patients, family members, and controls using the QIAGEN Gentra Puregene Blood kit following manufacturer's instructions.

Genome-Wide Genotyping, Linkage Analysis. Genome-wide genotyping was performed on the Illumina Human Core Genotyping Chip Array with > 250,000 SNPs. SNPs that were in high linkage disequilibrium were pruned using a threshold of LD > 0.05 in a sliding window of 50 SNPs. All SNPs were subjected to quality control with the following quality-control filters: per-SNP call rates > 95%, per-sample call rates > 95%, and MAF > 0.001 (PLINK software). Genotypes with Mendelian inconsistencies were excluded from the analysis (PEDCHECK). Genome-wide linkage was performed using MERLIN under the autosomal dominant model with disease allele frequency of 0.00003, phenocopy rate of 0.001, and penetrance of 50%.

Exome Sequencing. Exome sequencing for familial IgAN pedigree (Figure 1) was performed on the Illumina HiSeq 2000 platform using the Agilent Sure Select Human All Exon v.2 Kit following manufacturer's instructions. A total of 44 Mb of human exome was targeted, covering 98.2% of the CCDS database. For each capture experiment, 5 ug of genomic DNA was fragmented, linkers were ligated to the ends, and a library was prepared. Genomic DNA was annealed to capture probes and bound DNA was eluted, subjected to sequencing, and analyzed. The average read depth is 66X, and the D15 coverage is 86% (i.e., 86% of samples are covered by > 15 reads). All quality scores and metrics demonstrated excellent coverage and little bias in variant calling.
Exome sequencing for the second cohort of 418 IgAN patients and other CKD cases was performed on the Illumina HiSeq 2000 or 2500 platform and captured using the Roche Seq Cap EzMedExome or Roche Seq Cap EZ Exome V2 kit, as previously described (50).

Variant calling and annotation. Quality control was performed on Illumina raw reads using FASTQC and reads with a Phred quality score < 30 were excluded from further analysis. Filtered reads were aligned to NCBI Genome Reference Consortium Human build 37 using the BWA algorithm. Alignment and variant calling were performed using GATK. Called variants were annotated using Annovar and Seattle Seq. Variants were filtered and prioritized using an efficient bioinformatics pipeline. Variants with a low read depth were excluded, defined as variants with read depth <50x for both single nucleotide variants (SNV) and insertion-deletions (INDELS). Variants with a genotype quality score < 20 or a read depth of < 10x and allelic read depth of < 0.25 were excluded. Indels with a strand bias of < 200 and a read position rank sum of < -20 were excluded. Variants that were detected in high frequency (defined as a MAF >1%) in public databases such as in dbSNP, 1000 genomes and gnomAD v4.0 databases were excluded from analysis. Variants that were not shared among affected individuals in the same pedigree were also excluded. All variants that imparted a deleterious effect (nonsense, splice site, coding

insertion-deletions, and missense variants that are predicted to be damaging by PolyPhen2/SIFT and CADD) were prioritized.

Variant Validation and Segregation. Prioritized variants were validated using bi-directional Sanger Sequencing. Highly specific (evaluated by BLAST Expect value) primers were designed 200bp away from the site of variant to have an amplicon size of 500 -700 bp length, capturing the entire length of the exon in most cases. Primers (Supplemental Table 5) were designed to be 16-25 bp in length, have 50% GC nucleotide content, and a Tm of 60°C. PCR was performed using the following conditions on a Bio-Rad MyCycler Thermal Cycler: 95°C for 3 min followed by 36 cycles of 95°C for 30 s, 60°C for 30 s and 72°C for 1 min, and a 10-min incubation at 72°C. PCR amplicons were then used for Sanger sequencing. Sanger sequencing was serviced through Macrogen, Inc. Sequenced fragments were analyzed using Sequencher Analysis Software by aligning to the RefSeq FASTA sequence downloaded from the NCBI database. Presence of the variant confirmed by bi-directional Sanger sequencing was deemed as validated. Validated variants were further sequenced in the respective family members to test for segregation. Any variant present in all affected individuals in the family and segregating in the bloodline (absent in individuals married into the family) was considered as segregating. If unaffected individuals inherited the variant, the variant was retained under the presumption of incomplete penetrance. Any variant that met these criteria was considered as segregating. These segregating variants were prioritized for targeted re-sequencing. The segregating variant on *GALNT14* found on exon 10 in Ped-002 was further sequenced in 308 ethnically matched controls. Sequencing of the whole coding segments of the gene (15 coding exons in all the three isoforms, including two alternative exons in isoform 2, and 1 alternative exon in isoform 3), was conducted using Sanger sequencing in the familial IgAN cohort described above (totaling 68 patients). Any variant that was picked up through Sanger sequencing was tested for segregation when possible and tested for frequency in a large cohort of ethnically matched controls (n ≈ 300 controls/variant).

IgA1-producing cell lines. We used IgA1-secreting cell lines that were previously obtained by using limiting dilutions of Epstein-Barr Virus (EBV)-immortalized PBMC from patients with biopsy-proven IgAN and healthy controls, as previously described (30,32). Briefly, PBMCs from the donors were isolated from heparinized peripheral blood by Ficoll-Hypaque density gradient. To enrich for B cell population, we removed adherent cells through incubation in a plastic tissue-culture flask for 1 h at 37°C. T cells were removed by CD3 (panT) Dynabeads, as described (30,32). After a 30-d expansion to generate the first batch of immortalized B cells, we used a limiting-dilution method with several rounds of cloning and screening to generate IgA1-producing cell lines. Cell lines generated from four IgAN patients and four healthy controls were used for assessment of *GALNT* expression.

Assessment of GALNT expression. mRNA levels of 20 human *GALNTs* were determined by RealTime PCR using gene-specific primers (Supplemental Table 6) using previously published methods (30,32).

Determination of human IgA and Gd-IgA1. Serum IgA concentration was measured by capture ELISA (30,32). Gd-IgA1 was measured by a lectin ELISA with a GalNAc-specific lectin from *Helix aspersa* (now replaced by Sigma, Cat no: L6512) after enzymatic removal of sialic acid by neuraminidase, as previously described (30,32). The amount of IgA applied to the plates was normalized to 100 ng per well and the lectin binding was measured as optical density (OD) at 490 nm.

cDNA library and immunohistochemistry. Expression levels of *GALNT14* were measured in two human cDNA libraries from Clontech - the human Multiple Tissue panel and Immune system MTC I panel (Cat no: 636742 and Cat no: 636748). Gene expression was measured by RealTime PCR using gene-specific primers (Supplemental Table 5) with β-actin as an endogenous control.

Immunohistochemical staining of GalNAc-T14 was performed on paraffin-embedded tissue sections of human spleen, lymph node, and kidney, and on C57BL/6J mouse spleen, colon, and kidney after 10% formalin fixation. Slides were incubated with anti- GalNAc-T14 (Proteintech, Cat no: 16939-1-PBS) at 1:200 dilution, followed by biotinylated goat anti-rabbit IgG antibody (Vector Labs, Cat no: BA-1000-1.5) and developed with DAB substrate (Vector Labs, Cat no: SK-4100).

Mice. B6;129S5-Galnt14tm1Lex/Mmucd (*Galnt14-/-*, (MMRRC 032320-UCD)) mice were purchased from the Mutant Mouse Regional Resource Center. Mice were maintained in the barrier facility at Columbia University Irving Medical Center. All animal studies were approved by the Animal Care and Use Committee at CUIMC. Gene knockout was confirmed by RealTime PCR using *Galnt14*-specific and *Galnt14* isoform-specific primers (Supplemental Table 7) on wild-type, knock out, and heterozygous mice. There was no embryonic lethality.

Mouse aging experiments, IgA immunofluorescence. Aged mice (*Galnt14-/-*, *Galnt14-/+* and *Galnt14+/+*) were sacrificed at the 8-month and 12-month time points, and kidneys were collected and prepared for paraffin embedding in 10% formalin. Kidney tissue was prepared for cryo-sectioning in OCT medium (Tissue Tek). Paraffin-embedded sections were cut in 0.2 μm slices for light microscopy; slides were prepared and stained with hematoxylin and eosin. An additional slide of kidney tissue was stained with periodic acid-Schiff. Immunofluorescence (IF) was performed on 3-μm cryostat kidney sections using polyclonal FITC-conjugated antibodies to IgA (Dako, Cat no: F0204). IF staining intensity was graded from 0 to 3+ on a semi-quantitative scale and was scored as either presence or absence of IgA staining for statistical analysis.

Mouse DSS-induced colitis. DSS (s1) was used to induce acute colitis. On days 1-5, 2.5% DSS (MP Biomedicals mol wt ≈ 40,000 Da, MDL no: MFCD00081551) was supplemented in drinking water and provided ad libitum to G*alnt14-/-*, G*alnt14+/+*, and G*alnt14-/+* mice. Plain drinking water

was given to control mice. On days 6-8, DSS:H_2O mixture was replaced with plain drinking water for experimental mice. Kidney and colonic tissues were collected for microscopy on day 8.

Mucin staining and thickness measurements. Mucin staining was determined using Alcian Blue at pH 2.5 on deparaffinized colon sections according to protocol (s2). Mucin thickness was measured using ImageJ software on representative Alcian blue-stained sections of colon tissues of both *Galnt14+/+* and *Galnt14-/-*. Briefly, pixels were converted to micrometer using the scale bars on images that were captured at 600x magnification. Mucin thickness was then measured using the line drawing feature and calculating the width of visualized mucin along the luminal border.

Collection of plasma. Briefly, plasma was collected from heart bleeds into a Lithium Heparin tube (MiniCollect) and centrifuged at 1,500 g for 10 min; plasma was collected, aliquoted, and frozen at -80^oC until used.

Extraction of IgA from fecal samples. Fresh fecal pellets were suspended in 100 µl of PBS per 10 mg of feces. Pellets were vortexed and centrifuged at 600 g for the removal of large particles. Supernatants were removed and centrifuged at 6,000g to pellet the fecal bacteria; the supernatant was removed and considered to contain un-bound IgA. The pellet was re-suspended in 25 µl of PBS per 10 mg of starting material.

Extraction of IgA from the small Intestine. 10 ml of PBS was passed through the small intestine, via a catheter to wash out the IgA, bacteria, and debris. The eluant was vortexed and centrifuged at 600 g for the removal of large particles. Supernatants were removed and centrifuged at 6,000 g to pellet the fecal bacteria, and the supernatant was removed and considered to contain un-bound IgA. The pellet was re-suspended in 500 µl of PBS.

Extraction of IgA from the Peritoneal Cavity. 3 ml of ice-cold PBS was injected into the peritoneal cavity of mice, and the peritoneal cavity was massaged for 30-40 s. The PBS was removed from the peritoneal cavity and centrifuged at 300 g to pellet the cells, and the supernatant was aliquoted and frozen at -80°C until use.

Ex vivo cell culture. Cells were isolated from the spleen and the peritoneal cavity. Red blood cells were lysed using ACK lysing buffer and washed twice with complete tissue-culture medium. Lymphocytes were counted and cultured at a concentration of 5 x 10^{6}, in a 24-well plate for 6 days in a 37°C incubator, 5% CO_2, before the supernatants were removed from the cell culture. The supernatants were centrifuged at 300g for 10 min to remove any cells before transferred, aliquoted, and frozen at -80°C until use.

Measurement of immunoglobulins. IgA and IgG concentrations in the plasma, and mucosal compartments were measured using an anti-mouse IgA ELISA kit (Bethyl Laboratories Inc, Cat no: E99-103) or an anti-mouse IgG ELISA kit (Bethyl Laboratories Inc, Cat no: E99-131), following manufacturer's instructions. ELISA plates were read on a spectrophotometer (Power Wave XS, Bio-Tek) and the concentrations of IgA and IgG were calculated.

Assay for J-Chain-containing IgA. ELISA 96-well plates were coated with anti-J-Chain antibody (Proteintech, Cat no: 13688-1-AP). Plates were washed 3 times with the wash buffer (TBS with 0.05% of Tween20) and blocked for 60 min at room temperature. Plates were washed 3 times with the wash buffer, then serum samples (diluted 1:500 in wash buffer) were added to the plate and incubated for 90 min at room temperature. Plated were washed 5 times with wash buffer, and HRP-conjugated anti-mouse IgA was added to the plate and incubated at room temperature for

60 minutes. Plates were washed 7 times and developed using TMB for 12 min before stopping with H_2SO_4. ELISA plates were read on a spectrophotometer (Power Wave XS, Bio-Tek).

Flow cytometry of fecal derived bacteria. Bacteria isolated from the fecal pellets were re-suspended in 200 µl of flow buffer and blocked using anti-mouse Fc Block (anti-mouse CD16/CD32, BD Biosciences, clone 2.4G2), for 30 min on ice, before staining with IgA-FITC (Southern Biotech, cat no: 1040-02,) for 30 min on ice. The bacteria were washed twice in flow buffer and fixed in 250 µl fixation buffer (1% formaldehyde, 1 x PBS); the cells were analyzed using an LSR II flow cytometer (BD Biosciences) and FCS Express Flow software (V6, DeNovo Software) and Flow Jo software (10.8.1, Becton Dickinson & Company).

Flow cytometry of B cells. Single-cell suspensions were generated from the PBMC, spleen, peritoneal cavity, PP, mesenteric LN, mandibular LN, inguinal LN, lumbar LN, and popliteal LN. Cells were counted, and 1 x 10^6 cells were used for flow cytometric analysis of the isolated lymphocytes. Single-cell suspensions stained for viability (live/dead Zombie Yellow) were washed twice with flow buffer (1 x PBS, 2% FBS), re-suspended in 200 µl of flow buffer, and blocked using anti-mouse Fc Block (anti-mouse CD16/CD32, BD Biosciences, cat no: 553141), for 30 min on ice. The fluorescently labelled antibody cocktail was added to the re-suspended cells CD45-BV510 (BioLegend, Cat no: 103138), CD19-PE-Dazzel-594 (BioLegend Cat no: 115554), Dump (CD3, and CD14)-PerCP-eFluor-710 (eBioscience, Cat nos: 46-0032-82, 46-0141-82), IgD-BV650 (BioLegend, Cat no: 405721), IgA-FITC (Southern Biotech, Cat no: 1040-02), IgM – BV605 (BioLegend, Cat no: 406523), CD95-BV711 (BD Biosciences, Cat no: 740716), GL7-PE (BioLegend, Cat no: 144608), PNA-Cy5 (Vector Labs, Cat no: CL-1075-1), and incubated on ice for 30 min. Cells were washed twice using flow buffer and fixed in 250 µl fixation buffer (1% paraformaldehyde, 1 x PBS); cells were analyzed using an NovoCyte Penteon flow cytometer and FlowJo software (, Becton Dickinson & Company, Cat no: 10.8.1).

Adoptive transfer experiments. Splenocytes were isolated from the donor (*Galnt14-/-* and *Galnt14+/+* mice); red blood cells were lysed and prepared to a single-cell suspension. Spleens were washed twice in I x PBS (Ca- and Mg-free) before being resuspended in 1 ml of 1 x PBS. The isolated splenocytes were stained with Cell Trace Far Red (ThermoFisher Scientific, Cat no: C34564) at a 1:1000 dilution for 15 min at 37°C in the dark, before the dye was quenched using 10 ml of 1 x PBS and normal mouse serum for 5 min at 37°C in the dark. Cells were washed twice in 1 x PBS and 0.5% normal mouse serum. Cells were counted and resuspended to a final concentration of 3.5 x 10^{6} cells per 200 µl. Splenocytes from the donor mice were adoptively transferred to recipient mice via tail-vein injection; recipient mice were euthanized 24 hours post adoptive transfer; and cells were isolated from the circulation and the spleen, as described in the above section. To determine the presence of the adoptively transferred cells, the isolated cells were resuspended in 200 µl of flow buffer and blocked using anti-mouse Fc Block (anti-mouse CD16/CD32, BD Biosciences, Cat no: 553141), for 30 min on ice. The fluorescently labelled antibody cocktail was added to the re-suspended cells: CD45-AF488 (BioLegend, Cat no: 103122), CD19-APC-Fire (BioLegend, Cat no: 115558), CD3-BUV-395 (BD Biosciences, Cat no: 740268) and incubated on ice for 30 min. Cells were washed twice using flow buffer and fixed in 250 µl fixation buffer (1% paraformaldehyde, 1 x PBS); cells were analyzed using an LSR II flow cytometer (BD Biosciences) and FCS Express Flow software (V7, DeNovo Software).

Microbiome analysis. DNA extraction, 16S rRNA gene sequencing: DNA was extracted from mouse fecal pellets using a DNeasy PowerSoil Pro Kit with a 96-well plate format (Qiagen, Germany). The extraction process incorporated negative controls (HyPure™ Molecular Biology Grade Water, Cytiva) and positive controls which comprised defined bacterial communities (Zymo, CA). For DNA extracted from mouse fecal pellets, the V3V4 region of the 16S rRNA gene was amplified using primers with Illumina adapters. The resulting libraries were barcoded using

Illumina Nextera XT v2 indexes and sequenced on an Illumina MiSeq system with a v3 reagent kit (600 cycles) at a loading concentration of 8 pM and included a 20% phiX spike-in.

16S rRNA Microbiome Analysis: The 16S rRNA sequences were processed and analyzed using the DADA2 (version 1.12.1) pipeline (s3) and R version 4.1.0. DADA2 facilitated quality filtering, trimming, error correction, exact sequence inference, chimera removal, and generation of the amplicon sequence variant (ASV) table. No ambiguities were allowed in the overlap region, and default settings were used in the DADA2 filterAndTrim() function, with parameters set as follows: truncLen=c(250,240), trimLeft=c(5,5), maxN=0, maxEE=c(2,2), and truncQ=2. Chimeric reads were identified by consensus across samples using the removeBimeraDenovo() function in DADA2. All samples met the minimum requirement of 7,500 reads after quality filtering to be included in this analysis. Phylogenetic trees of all ASV sequences were constructed using the MAFFT and FastTree modules in QIIME2. Taxonomic classification was conducted with a native naïve RDP Bayesian classifier against the Silva version 138 database (s4).

Statistical analysis for microbiome: Genotypic data were collected, screened, and summarized using descriptive statistics for the entire cohort. The data were then imported into R version 4.1.0 for further analysis. Taxa with a mean relative abundance below 0.0005 across all samples were excluded from the dataset prior to all analyses. Alpha-diversity indices, such as Shannon and Chao and Principal Coordinates Analysis (PCoA) using the Bray, UniFrac, and weighted UniFrac distance matrices, were computed with the phyloseq package (version 1.36.0) (s5). The alpha-diversity (a measure of species richness and evenness within a sample) differences between groups were assessed using robust linear regression to account for the non-constant variance in the data. Beta-diversity (a measure of differences in microbial community composition between samples) was examined through permutational multivariate analysis of variance (PERMANOVA), a non-parametric method that evaluates differences in sample centroids. The p-values obtained were adjusted using the Benjamini-Hochberg method to control the false discovery rate. All analyses controlled for the influence of sex.

Statistical analysis. Graphs and statistics were done in Prism (V9 for macOS). The data was initially analyzed for the distribution (normal or not normal) and statistical differences between two groups was identified using an un-paired t-test, or a Mann-Whitney test (* $P < 0.05$, ** $P < 0.01$, *** $P < 0.005$, and **** $P < 0.001$). Data in scatter plots represents the mean ± the standard deviation.

Data availability. Values for all data points in graphs are reported in the Supporting Data Values file. Further requests for data should be directed to the corresponding authors.

References:

50. Groopman EE, Marasa M, Cameron-Christie S, Petrovski S, Aggarwal VS, Milo-Rasouly H, et al. Diagnostic Utility of Exome Sequencing for Kidney Disease. *N Engl J Med.* 2018.

30. Suzuki H, Moldoveanu Z, Hall S, Brown R, Vu HL, Novak L, et al. IgA1-secreting cell lines from patients with IgA nephropathy produce aberrantly glycosylated IgA1. *J Clin Invest.* 2008;118(2):629-39.

32. Suzuki H, Raska M, Yamada K, Moldoveanu Z, Julian BA, Wyatt RJ, et al. Cytokines alter IgA1 O-glycosylation by dysregulating C1GalT1 and ST6GalNAc-II enzymes. *J Biol Chem.* 2014;289(8):5330-9.

s1. Perse M, and Cerar A. Dextran sodium sulphate colitis mouse model: traps and tricks. *J Biomed Biotechnol.* 2012;2012:718617.

s2. Elderman M, Sovran B, Hugenholtz F, Graversen K, Huijskes M, Houtsma E, et al. The effect of age on the intestinal mucus thickness, microbiota composition and immunity in relation to sex in mice. *PLoS One.* 2017;12(9):e0184274.

s3. Callahan BJ, McMurdie PJ, Rosen MJ, Han AW, Johnson AJ, and Holmes SP. DADA2: High-resolution sample inference from Illumina amplicon data. *Nat Methods.* 2016;13(7):581-3.

s4. Quast C, Pruesse E, Yilmaz P, Gerken J, Schweer T, Yarza P, et al. The SILVA ribosomal RNA gene database project: improved data processing and web-based tools. *Nucleic Acids Res.* 2013;41(Database issue):D590-6.

s5. McMurdie PJ, and Holmes S. phyloseq: an R package for reproducible interactive analysis and graphics of microbiome census data. *PLoS One.* 2013;8(4):e61217.
